# Giant Decrease in Interfacial Energy of Liquid Metals by Native Oxides

**DOI:** 10.1002/adma.202406783

**Published:** 2024-10-10

**Authors:** Woojin Jung, Man Hou Vong, Kiyoon Kwon, Jong Uk Kim, S. Joon Kwon, Tae‐il Kim, Michael D. Dickey

**Affiliations:** ^1^ School of Chemical Engineering Sungkyunkwan University (SKKU) Suwon 16419 South Korea; ^2^ Department of Chemical and Biomolecular Engineering North Carolina State University (NCSU) Raleigh 27695 USA

**Keywords:** Eutectic Galluim‐Indium (EGaIn), galinstan, gallium, interfacial energy, liquid metal (LM)

## Abstract

Native oxides form on the surface of many metals. Here, using gallium‐based liquid metal alloys, Johnson‐Kendall‐Roberts (JKR) measurements are employed to show that native oxide dramatically lower the tension of the metal interface from 724 to 10 mN m^−1^. Like conventional surfactants, the oxide has asymmetry between the composition of its internal and external interfaces. Yet, in comparison to conventional surfactants, oxides are an order of magnitude more effective at lowering tension and do not need to be added externally to the liquid (i.e., oxides form naturally on metals). This surfactant‐like asymmetry explains the adhesion of oxide‐coated metals to surfaces. The resulting low interfacial energy between the metal and the interior of the oxide helps stabilize non‐spherical liquid metal structures. In addition, at small enough macroscopic contact angles, the finite tension of the liquid within the oxide can drive fluid instabilities that are useful for separating the oxide from the metal to form oxide‐encased bubbles or deposit thin oxide films (1–5 nm) on surfaces. Since oxides form on many metals, this work can have implications for a wide range of metals and metal oxides in addition to explaining the physical behavior of liquid metal.

## Graphical abstract

The native oxide of liquid metal (LM) lowers the interfacial energy from 700mN/m (bare metal) to ∼10 mN/m due to the anisotropy of the oxide: metallophilic inward‐facing, and hydrophilic outward‐facing interfaces. This decrease helps explain the unusual interfacial behavior of LM, such as adhesion to surfaces and dewetting of the metal from the oxide, including from an oxide‐encased air bubble.

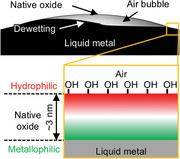

## 1 Introduction

Metals have strong metallic bonds that lead to enormous surface energy, usually a few hundred mN/m or higher.^[^ ^1^ ^]^ For example, liquid gallium (the focus of this article) has a surface tension of 724 mN m^−1^. Yet most metals, including gallium, react with air to form very thin (nm thickness) native oxides on their surface. These oxides are critical for protecting metals, such as aluminum and stainless steel, against further oxidation.

**Scheme 1:**
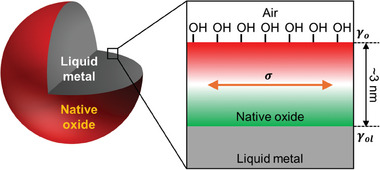
Native oxide of liquid metal and its two interfaces. The surface energy of the outward‐facing interface, the interfacial energy of the inward‐facing interface, and the mechanical tension of the native oxide are denoted as γ_*o*_, γ_*ol*_, and σ, respectively.

The presence of such oxides on metals also has several implications for interfacial behavior. First, it creates a solid, mechanical coating that can sustain mechanical tension along the surface (σ, **Scheme** **1**).^[^ ^2^ ^]^ Second, native oxides generate two new interfaces: metal/oxide and oxide/air (Scheme 1).^[^ ^3^ ^]^ Importantly, the outward‐facing interface (i.e., oxide/air) is fully oxidized and typically terminates with polar hydroxyl groups.^[^ ^4^ ^]^ We depict this outward‐facing interface as red throughout the manuscript, including Scheme 1. Yet, the inward‐facing interface (i.e., oxide/metal) does not form hydroxyl groups; instead, the inner‐most oxygen atoms terminate by bonding to metal atoms at the interface,^[^ ^5^ ^]^ thereby forming a metallophilic^[^ ^6^ ^]^ interface.^[^ ^7^ ^]^ We depict this inward‐facing interface as green throughout the manuscript, including Scheme 1. Thus, the native oxide is asymmetric and therefore holds some analogies with molecular surfactants that assemble at liquid interfaces.

Conventional molecular surfactants are also asymmetric since they often consist of polar or charged head groups that orient toward the more polar fluid (e.g., water) while nonpolar tails (e.g., alkyl chains) orient toward the less polar surrounding medium (e.g., air or oil). This asymmetry enables the surfactant to orient at interfaces and decrease the interfacial tension.^[^ ^8^ ^]^ Analogously, the asymmetry of native oxides offers the possibility of them being incredibly effective “surfactants” for lowering the interfacial energy of metals. Unlike molecular surfactants, which need to be externally synthesized and added to the liquid, oxides form spontaneously via in situ reactions only at the interface without altering the bulk purity of the metal.

Measuring the extent of the surface activity of oxides at the buried oxide/metal interface is experimentally difficult to probe. Most methods for determining interfacial energy rely on either direct measurement of the force (e.g., Wilhelmy plate or Langmuir–Blodgett trough) or indirect measurements through drop shape analysis.^[^ ^9^ ^]^ In either case, it is difficult to decouple the mechanical effects of the solid native oxides from the interfacial energy of the metal inside its own native oxide. For example, prior studies of molten metals suggest that oxide species can lower the tension of the metal as a function of the partial pressure of oxygen.^[^ ^10^ ^]^ The pendant drop shape–which represents a balance between gravitational forces and interfacial forces–determines the interfacial energy in such measurements. Yet, the measurements fail to be meaningful once a solid, conformal oxide forms on the surface since the oxide mechanically encases the liquid (Table S1, Supporting Information). Thus, the extent to which the interior of native oxides lowers the tension of the metal has been challenging to measure. We sought to measure interfacial energy at the metal/interior‐oxide interface (γ_*ol*_, Scheme 1) and thereby decouple the effect of the mechanical tension of the oxide, σ, on such interfacial measurements.^[^ ^11^ ^]^

We chose molten gallium (Ga) for this study because its melting point (29.8 °C) is experimentally easy to access and rapidly forms a thin, passivating native oxide similar to aluminum, a commercially important metal. We also include EGaIn and Galinstan in this study since they are also liquid metals that form gallium oxide at their surface. We show that these native oxides formed in air can significantly lower the interfacial energy of the metal from 724 to ≈10 mN m^−1^. This change is by far the largest reported for a surfactant; in contrast, adding conventional molecular surfactants to bare liquid mercury (Hg) only lowers the surface tension from 485 to 424 mN m^−1^.^[^ ^12^ ^]^ Relative to conventional surfactants, native oxides have a high density dictated by strong chemical bonding^[^ ^3^ ^]^ (whereas conventional surfactants pack on surfaces with a lower density limited by entropy, which limits the surface pressure responsible for lowering the surface tension.^[^ ^8^ ^]^) Oxides are also stable to high temperatures, making them truly unique from conventional surfactants. While native oxides cannot flow like surfactants (e.g., they wrinkle under compression), they can break and reform readily in response to tensile strain in the presence of oxygen, ensuring that surfaces remain covered with oxide.

We note the distinction of this work from prior studies that report that electrochemical oxidation of liquid metal can lower the net effective tension based on drop‐shape analysis.^[^ ^13^ ^]^ The electrochemical mechanism for lowering tension is distinct from the work here. Key differences include the following: Electrochemical oxidation 1) generates charged species as well as oxidative stress at the interface that would not occur in air,^[^ ^14^ ^]^ 2) produces a thinner oxide layer (likely a monolayer) than that formed in air (≈3 nm),^[^ ^15^ ^]^ and 3) creates chemical species (gallium hydroxide, GaOOH and Ga(OH)_3_) that are different from those that form in air (Ga_2_O_x_ where x≈3). In addition, electrochemical oxidation occurs in electrolytes that can dissolve and hydrate the oxide, whereas the oxide that forms in air is static and stable.^[^ ^13^ ^]^ Finally, studies of the electrochemical mechanism focus on the net effective tension between metal and electrolyte that is determined by the compressive stress resulting from the continuous oxidation reaction at the interface,^[^ ^14^ ^]^ and do not analyze how and to what extent the gallium hydroxide (GaOOH and Ga(OH)_3_) is asymmetric. The purpose of this paper is to focus on the interface between the interior of the native oxide and the metal, the asymmetricity of the native oxide, and predicting the interfacial behavior of the liquid metal with the native oxide.

The ability of the oxide to lower the tension of the metal at the interface with the interior of its own oxide has multiple implications for the fluidic behavior of liquid metal. Bare liquid metal is difficult to micro‐pattern since it has an enormous surface tension that causes the molten metal film to adopt spherical shapes. However, with the oxide layer, it is well known that stable non‐spherical shapes such as cylinders, and films can be formed by a number of methods, including injection into microchannels, direct‐write printing, and stencil printing.^[^ ^16^ ^]^ In addition to creating a solid shell around the metal, we reason that by lowering the tension of the metal itself, the oxide also enhances the stability of microstructures by mitigating the destabilizing Laplace pressure of the liquid. To illustrate this principle, we identify conditions in which the Laplace pressure is sufficient for the metal to dewet from its oxide, thereby enabling separation of the oxide from the metal.^[^ ^17^ ^]^

In addition, the oxide also has obvious implications for adhesion to surfaces. While the interior of the oxide lowers the tension of the metal, the oxide simultaneously creates an outward‐facing interface that dramatically changes the interfacial interactions with other surfaces.^[^ ^4^, ^8^ ^]^ Without the oxide, the bare metal forms primarily metallic bonds with surfaces, whereas with the oxide it can form more common interactions such as van der Waals forces, Lewis acid‐base interactions, and hydrogen bonding. Thus, this study of native oxides provides interesting new insights into an overlooked class of “surfactants” with unprecedented surface activity and implications for stabilizing molten films, micropatterning liquid metals, adhering metals to surfaces, and separating oxides from metals (as a means to deposit oxides at ambient conditions or create oxide bubbles).

## 2 Results

**Figure 1:**
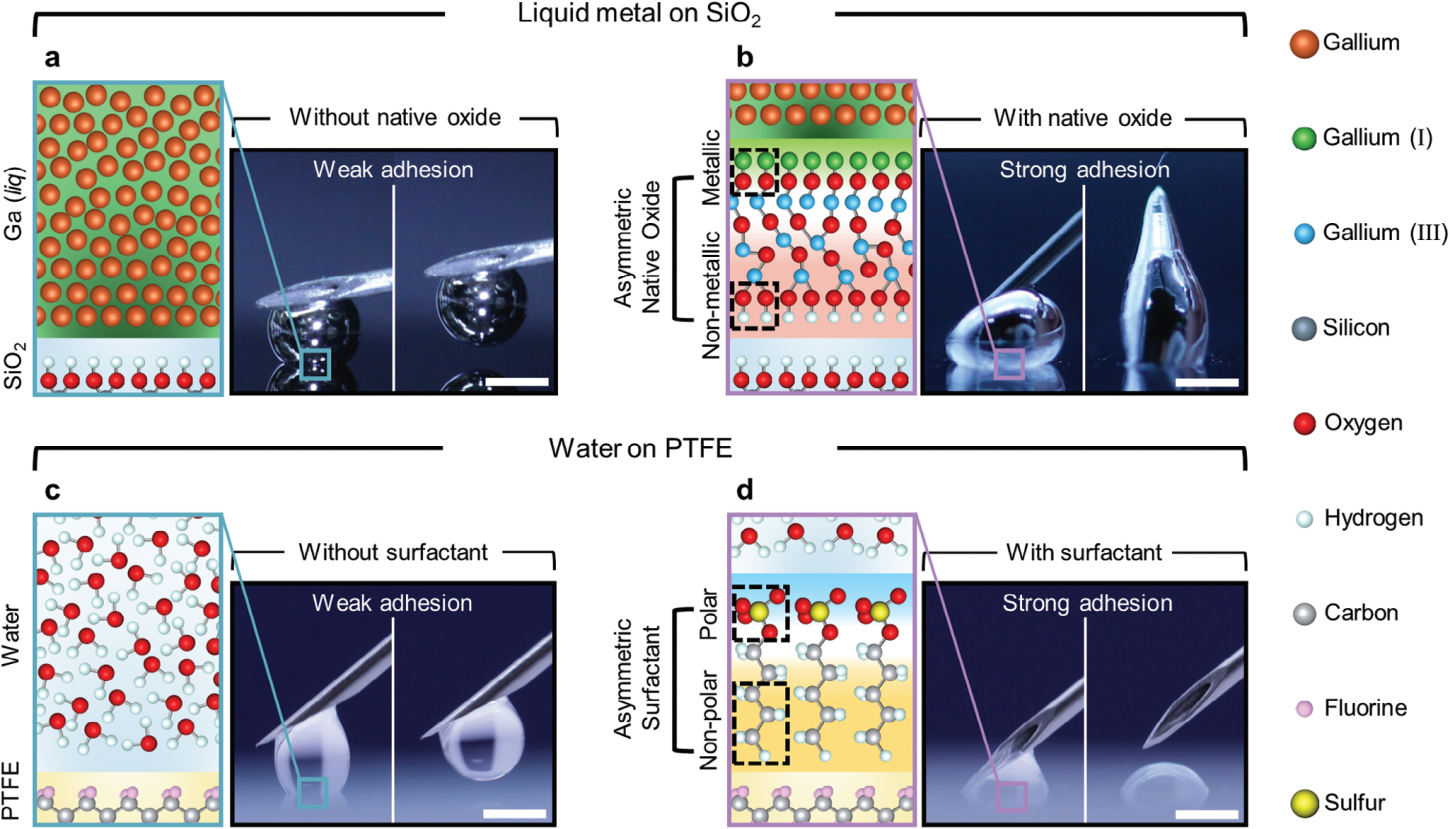
Asymmetric native oxides significantly lower the interfacial energy of metal and affect adhesion. a) Bare LM (gallium) does not wet the hydroxy‐terminated exterior of the native silicon oxide on a Si wafer. Oxygen and H_2_O concentrations were kept below 1 ppm in a glove box to prevent oxidation of the liquid metal surface. We polished the needle to remove the native oxide of the needle which improves the metallic adhesion between the LM and the metal suface of the needle. b) In contrast, LM wets the interior of its own oxide, while the exterior of the oxide adheres to the SiO_2_. c,d) Likewise, pure water (c) does not adhere to a hydrophobic substrate, poly(tetrafluoroethylene)(PTFE), but does with a surfactant (sodium dodecyl sulfate, 0.5 wt.%) (d). The asymmetry of the native oxide layer in (b) is analogous to the asymmetry of the surfactant in (d), yet is two orders of magnitude more effective at lowering interfacial energy. Scale bars = 1 mm.

Bare Ga (without oxide skin) has an enormous surface tension, γ_*l*_, of 724 mN m^−1^.^[^ ^18^ ^]^ This value is similar in magnitude to other molten metals and an order of magnitude larger than water (72 mN m^−1^) and organics (20–50 mN/ m^−1^).^[^ ^19^ ^]^ The enormous tension of bare liquid metals (γ_*l*_) arises from the significant contribution of both dispersive intermolecular interactions ($\gamma_{l}^{d}$) and metallic bonding ($\gamma_{l}^{m}$). (Supporting Information, Calculation of interfacial energy and Hamaker constant of materials.) When a bare metal contacts a surface, a new interfacial energy arises (γ_*ls*_). The value of γ_*ls*_ depends on the composition of the surface: the metallic component of the surface tension can interact strongly with other metallic surfaces, while dispersive components interact universally. Since dispersive interactions are a small fraction of the overall tension of bare metals, the adhesion to most non‐metallic surfaces is weak.^[^ ^20^ ^]^ Consequently, bare droplets of liquid metal (LM) without a native oxide (Figure S1a, Supporting Information) do not adhere to non‐metallic surfaces (**Figure** **1**a).

In contrast, it is well‐known that exposing liquid Ga to air completely changes its behavior (Figure 1b). The surface of Ga readily reacts with oxygen to form a native oxide skin that is 2–3 nm thick (Figure S1b, Supporting Information). The exposed surface switches from being metallic to being an oxide. Importantly, the interior interface of the oxide contains suboxide species that terminate with Ga atoms exhibiting strong affinity to the LM, whereas the exterior of the oxide is fully oxidized, terminating with hydroxyl groups. Thus, the oxide is asymmetric: It has a metallic nature at the interior interface, and a hydrophilic nature at the exterior surface (Scheme 1; Figure 1). Such an interpretation is consistent with X‐ray photoelectron spectroscopy (XPS) measurements of the oxide, which suggest it is primarily in Ga(III) oxidation state (Ga_2_O_3_) but has a small amount of Ga(I) closer to the metal interface.^[^ ^7^ ^]^ This atomic layer of Ga(I) still has free electrons that promote metallic adhesion.^[^ ^6^ ^]^ Simultaneously, the exterior of the oxide can promote short‐range polar adhesion with non‐metallic polar surfaces due to the hydroxyl group. Consequently, the oxide‐coated metal readily adheres to smooth substrates (Figure 1b).

The asymmetric native oxide skin has similarities to a conventional surfactant, which also promotes the adhesion between polar and non‐polar phases by its asymmetric molecular structure (Figure 1c,d). For example, pure water does not adhere to the surface of poly(tetrafluoroethylene) (PTFE) (Figure 1c). In contrast, dissolved surfactant (sodium dodecyl sulfate, 0.5 wt.%) improves the adhesion of water to the PTFE surface (Figure 1d). The surfactant lowers the interfacial energy between the liquid and the substrate, while the polar head group of the surfactant strongly adheres to the water molecules. Consequently, the surfactant layer encasing a droplet of water promotes the adhesion between the water and the hydrophobic surface (Figure 1d). Note that we kept the Bond number (*Bo*  = ρ*gL* ^2^/γ , here ρ, *g*, *L*, and γ are density, gravitational acceleration, characteristic length, and surface tension of the droplet) of the droplets in Figure 1 below 0.1 to minimize the effect of gravity.^[^ ^21^ ^]^

We emphasize the premise of this work and the challenge it presents: the value of interfacial energy between the interior of the native oxide and liquid metal (γ_*ol*_) must be measured with the metal encased in a film of solid oxide. For this reason, conventional measurements of interfacial energy cannot be implemented since the mechanical effects of the oxide must be decoupled from γ_*ol*_. In essence, we sought to measure directly the value of γ_*ol*_ inside a “sac” of its own oxide.

**Figure 2:**
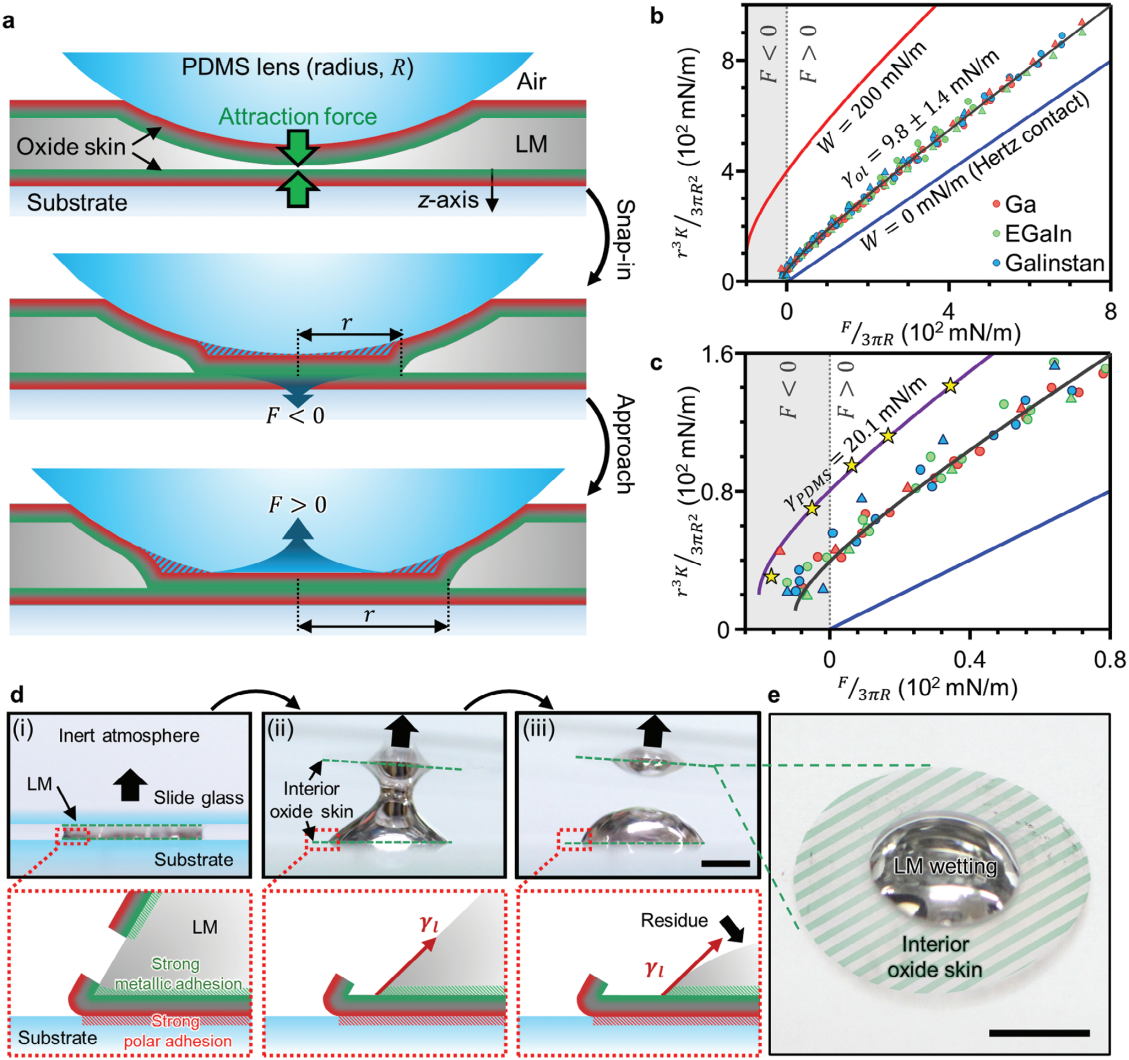
Interfacial energy measurement between the interior oxide skin and LM by the JKR method and adhesion behavior of LM to the interior of its native oxide skin a) Schematic of the JKR method that measures the radius (r) of the contact area between a silicone lens and a substrate (here, a puddle of liquid metal on a rigid substrate) versus the applied force (F). The asymmetry of the oxide skin is denoted as red (exterior: dispersive, polar), and green (interior: dispersive, metallic). b) Using the JKR method, the best‐fit theoretical line indicates a very low interfacial energy (γ_*ol*_ =  9.8 ± 1.4 *mN*/*m*). The red and blue traces show hypothetical values if γ_*ol*_was 0 or 200 mN m^−1^, respectively. The circles and the triangles represent fused silica substrates and PDMS substrates, respectively. The range from −24 to 80 mN m^−1^ of *F*/3π*R* of (b) is magnified in c) to show how sensitive the technique is to tension. Yellow stars represent the JKR adhesion result of a PDMS lens on a PDMS substrate without any liquid metal to confirm the method is giving reasonable results. d) Two glass slides compress a droplet of LM (gallium) with its native oxide. Separating the slides in an inert atmosphere (less than 1 ppm of O_2_ and H_2_O) breaks the oxide, exposes the metal, and allows it to flow freely. e) Rather than bead up, the LM wets the interior of its oxide skin (green hatched area). Scale bars = 5 mm.

To measure γ_*ol*_, we adopted the JKR (Johnson—Kendal–Roberts) adhesion theory, which is an established way to measure surfaces with low interfacial energy.^[^ ^22^ ^]^ In this experiment (Figure S4, Supporting Information), an elastic polydimethylsiloxane (PDMS) lens with a radius *R* presses against a puddle of LM encased on the top and bottom by its native oxide. The metal is therefore within a “sac” of its own oxide in which the metal is only in direct contact with “interior oxide” (distinguished using green shading in the figures). As the lens approaches the underlying rigid glass substrate, the attraction between the interior of the top and bottom oxide layers snaps the surfaces together (**Figure** **2**a). The snap‐in is apparent from the sudden drop in the force acting on the PDMS, denoted by a black arrow in Figure S5a–c (Supporting Information). All three LMs tested show similar force (*F*) in a range of −7.5∼−6.6 dynes at the moment of snap‐in (Figure S5a–c, Supporting Information). Pressing the lens further toward the substrate causes the lens to deform (Figure 2a) and the force to increase. It also increases the radius (*r*) of contact area between the lens and the substrate (Figure 2a; Figure S5d–f, Supporting Information). This area represents the competition between the attraction force between the two layers of “interior oxide” and the repulsion force from the elasticity of the sphere as it gets pushed toward the substrate. Measuring both the contact radius, *r*, and corresponding force, *F* we can readily calculate the adhesion energy (Figure S5g–l, Supporting Information, JKR adhesion).

Using the technique described in Figure 2a, we tested three different Ga‐based liquid metals on both fused silica and PDMS, as reported in Figure 2b. Hertz theory (blue line), which only considers mechanical properties and neglects interfacial energy, does not match the data. In contrast, JKR theory, which considers both interfacial energy and mechanical properties, fits all the data well and gives remarkably low interfacial energy, γ_*ol*_ =  9.8 ± 1.4 mN/m. Different substrates do not affect these results. This indicates that the long‐range force (dispersive interaction) arising from the PDMS lens and substrate is negligibly small. For comparison, we repeated the same measurement on a PDMS substrate without LM. PDMS has been previously reported to have a surface energy (γ_*PDMS*_) 21.6 mN m^−1^.^[^ ^22d^ ^]^ As shown in Figure 2c, an energy of 20.1 mN m^−1^ curve (purple line) fits the PDMS data, which gives further confidence to the values obtained by this method. We note that although the liquid gallium is slightly supercooled at room temperature relative to its melting point of 29.8 °C, the difference in tension measured at room temperature versus 29.8 C is expected to be negligible.^[^ ^18^, ^23^ ^]^

To illustrate the role of the oxide on adhesion, we pressed a droplet of LM between two glass slides (Figure 2d). The exterior of the oxide of the LM (depicted as red) adheres to both slides, where the dispersive and polar contributions lead to strong adhesion.^[^ ^24^ ^]^ Separating the glass slides in an inert environment causes the capillary break‐up of the LM into two hemispherical drops (one on each slide) due to the high tension of the exposed metal. Interestingly, the metal hemispheres assume a low contact angle, leading to an insightful result. Bare LM has an enormous surface energy and does not wet the exterior surface of oxides, such as the native oxide of silicon wafers (Figure 1a), Ga_2_O_3_, and even the native oxide of LMs (Figure S2, Supporting Information). Yet, the hemispherical LM remains wetted to the interior of its own oxide skin adhered to the substrate. Rather than remain pinned to the perimeter (black dashed line), the LM retracted partially (Figure 2e). Importantly, it does not “bead up”, but instead, forms favorable interactions between the metal and the interior oxide. This observation implies a low interfacial energy between the interior of the oxide and the metal, γ_*ol*_. In contrast, bare LM does not adhere to the exterior surface of the oxide skin (Figure S3, Supporting Information). These simple experiments provide evidence that the oxide skin has an asymmetric molecular structure like a surfactant.

Despite the extremely small γ_*ol*_, the tension of the oxide skin plays a significant role in the interfacial behavior of LMs. Prior studies show that the oxide breaks (yet rapidly reforms) when applied tensile forces exceed the critical surface yield stress of 300–700 mN m^−1^.^[^ ^2^, ^25^ ^]^ The range of values likely reflects the fact that measurements of mechanical failure are inherently noisy, geometry dependent (e.g., shear vs tension), and subject to hysteresis associated with sample handling that could cause wrinkles in the oxide. To confirm that γ_*ol*_ is much smaller than the equilibrium tension of the oxide on a puddle of metal (σ_*E*_), we used a Wilhelmy method for three LM alloys (Figure S6 and Movie S1, Supporting Information). The method involves gently conforming the external oxide to a PDMS lens while measuring the tensile force it exerts on the lens. The average tension of each sample was similar, ranging from 370 to 410 mN m^−1^ (Figure S7b, Supporting Information) similar range expected from previous measurements.^[^ ^2^, ^26^ ^]^

**Figure 3:**
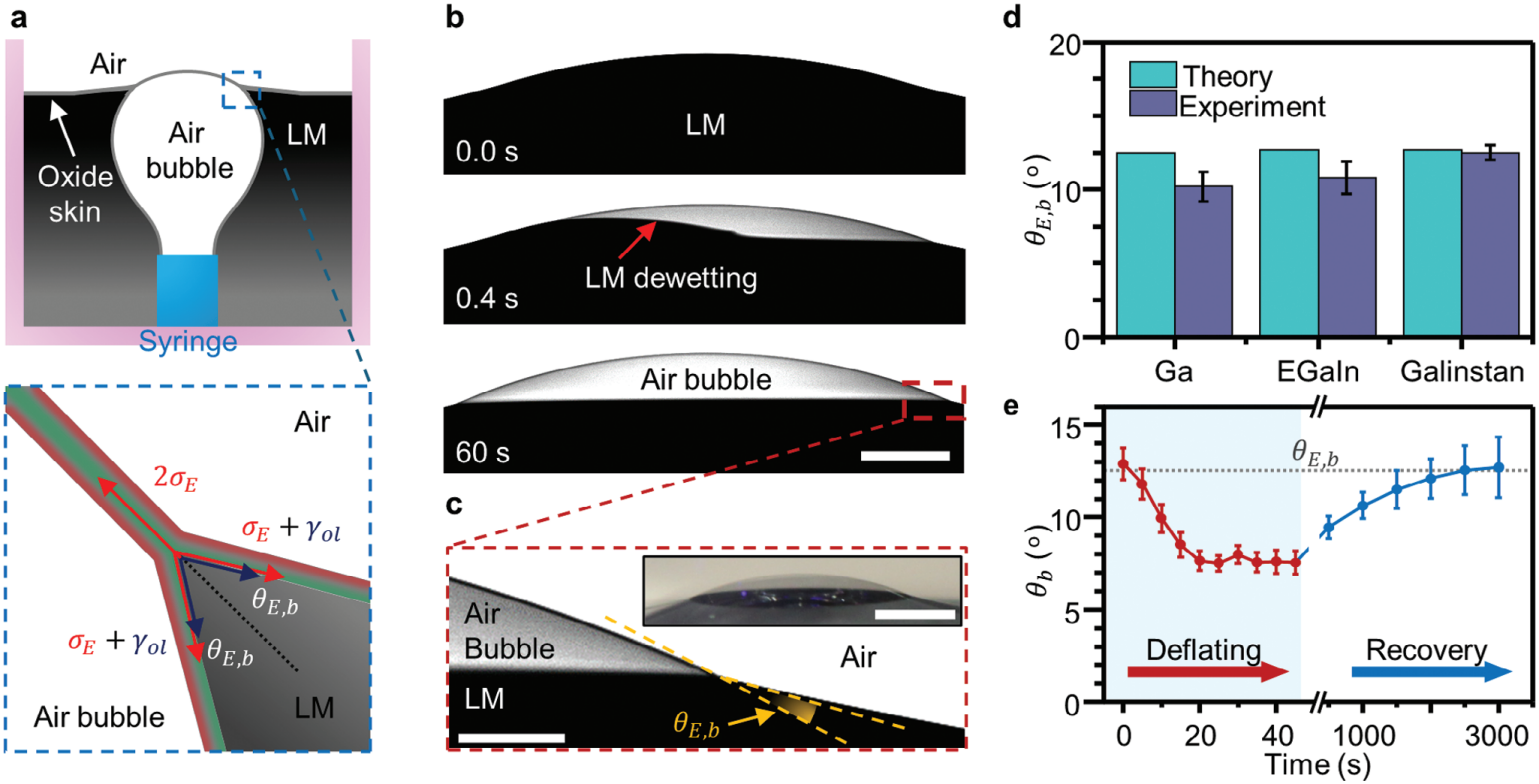
Spontaneous dewetting of LM from an oxide‐encased bubble. a) Liquid metal spontaneously dewets between two interior oxide skins. One skin forms on the surface of the LM bath and the other on an air bubble that rises buoyantly within the LM. The compositional asymmetry of the oxide skin is denoted as red (exterior: dispersive, polar), and green (interior: dispersive, metallic). The subscript *E* and *b* represent equilibrium, and bubble, respectively. b) Photographs of LM (gallium) dewetting from an oxide‐encased air bubble. The red arrow identifies the liquid metal at the dewetting front between the two interior oxide layers. c) The contact angle between the oxide‐encased air bubble and liquid metal (inset: Perspective view of the oxide‐encased air bubble, which remains stable.) d) Comparison between the theoretical equilibrium value of the contact angle θ_*E*,*b*_ and the experiment. e)The contact angle θ_*b*_ recovers to θ_*E*,*b*_ after the deflation of the bubble from its equilibrium state. Scale bars = (b) 500 µm, (c) 200 µm, (c, inset) 1 mm.

We corroborated the interfacial values by studying fluid instabilities induced by injecting a bubble of air inside of a bath of LM to form contact between two interior layers of oxide (**Figure** **3**). An oxide skin forms on both the bubble (Figure 3a) and the exterior surface of the LM bath. As buoyancy forces cause the bubble to rise, the two interior surfaces of the oxide (as indicated using green coloration) come into contact. Based on the snapping behavior reported in Figure 2, we expected these oxide surfaces to form intimate contact. Interestingly, once they come into contact, the LM between these two oxide layers spontaneously dewets (Figure 3b). Although the dewetting initiates in less than seconds, the metal continues to withdraw for several minutes until it reaches an equilibrium state (Figure 3b; Movie S2, Supporting Information). This confirms an important implication of the non‐zero γ_*ol*_ value: there exists an equilibrium contact angle of the metal within its own oxide as long as the liquid metal is not mechanically constrained by the oxide shell. The resulting oxide bubble (radius 1–2 mm) remained stable and only failed in response to a mechanical disturbance despite being encased by a membrane that is only a few nm thick (Figure S8, Supporting Information).

**Figure 4:**
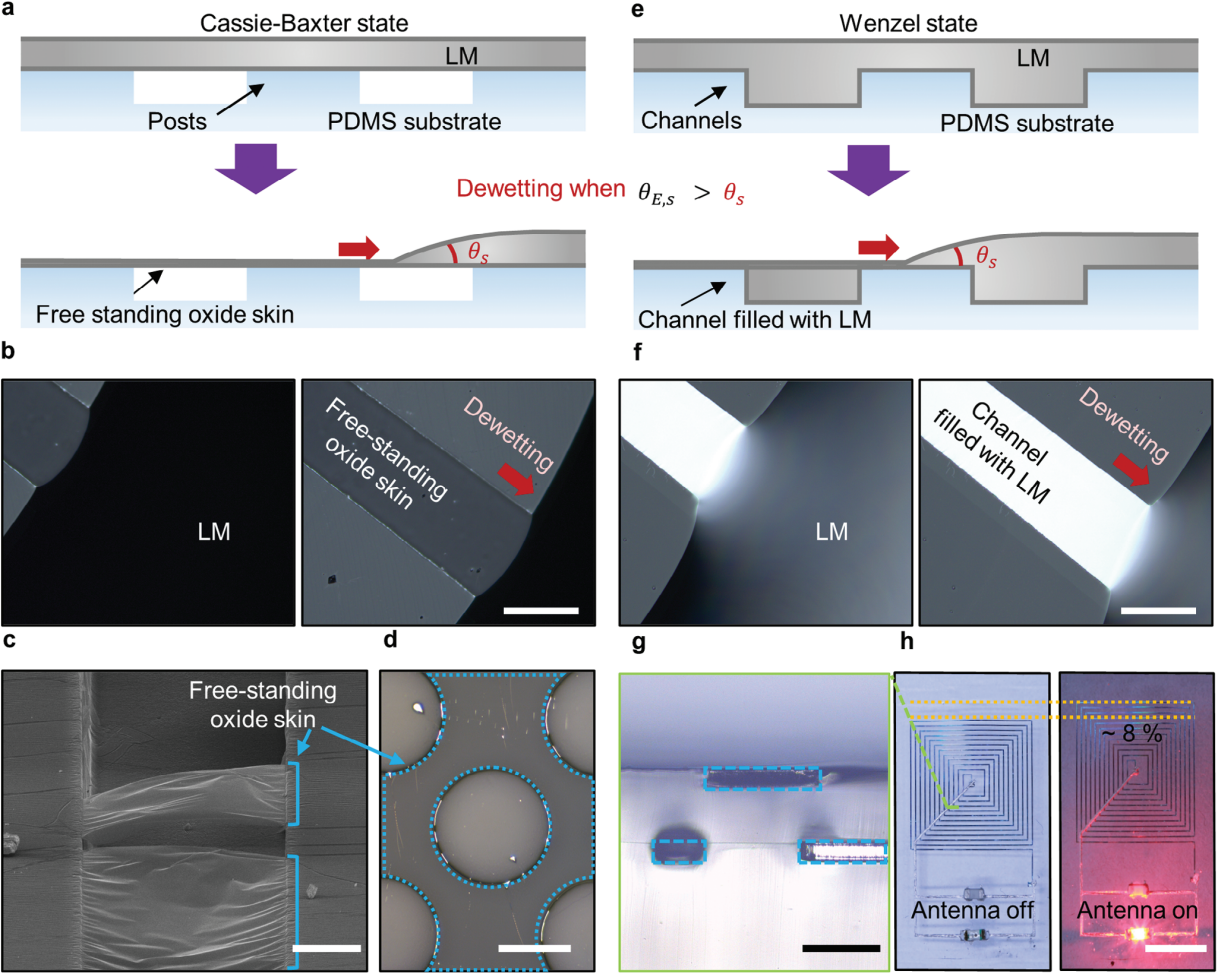
Applications of dewetting of LM from its own oxide on a structured surface. a–d) Dewetting of LM when it has not infiltrated surface topography, i.e., the Cassie–Baxter state. a) Schematics illustrating LM dewetting to leave only free‐standing oxide skin suspended over the topography. b) LM dewetting a PDMS surface with a linear groove. c) SEM image of the oxide skin suspended over the groove. d) Optical micrograph of the oxide skin suspended by circular PDMS posts. The darker area denoted by blue dashed lines is the suspended oxide skin. e–g) Dewetting of LM when it has infiltrated surface topography, i.e., the Wenzel state. e) Schematics illustrating LM dewetting the top plane of the substrate but not the grooves when it is in the Wenzel state. f) LM dewetting a PDMS surface with a linear groove. g) Cross‐sectional view of grooves filled with LM using this method. h) A stretchable near field communication (NFC) antenna consisting of LM connected to an LED, encased in PDMS. Scale bars, = (b, f, g) 200 µm, (c) 10 µm, (d) 100 µm, (h) 500 µm.

The bottom image of Figure 3a describes the balance of forces and energy on the bubble. Since the interface between the collapsed oxide skins is identical, its interfacial energy is assumed to be zero. Given the symmetry of the system, it is valid to assume that the two angles (θ_*E*,*b*_) are the same at the triple contact point. We measured the angle between the oxide skin on the liquid metal side and the collapsed oxide membrane (θ_*E*,*b*_) to be in the range of 10–13°. (Figure 3c) We also predict the theoretical angle using the previously measured γ_*ol*_and σ_*E*_values (Supporting Information, equilibrium liquid metal puddle). As shown in Figure 4d, the difference of the angle between the theory and experiment was less than 2°. We deflated the bubble by reducing the pressure inside of the syringe. During deflation, the contact angle (θ_*b*_) initially decreases (Figure 3e), presumably due to the decrease in tension of the oxide encasing air bubble. However, the angle slowly returns to the equilibrium value within ≈50 min after deflation.

The implication of Figure 3 is that under certain conditions, liquid metal can dewet the interior of its own oxide. The liquid metal starts dewetting when two interior oxide interfaces contact and the contact angle is lower than 18°–20°. This occurs by forming an air bubble inside bulk LM (Movie S2, Supporting Information), or by scratching a puddle of liquid metal (Movie S4, Supporting Information). The exact angle for different conditions can easily be predicted theoretically (Supporting Information, equilibrium liquid metal puddle) In turn, we can utilize this phenomenon to control the wetting or dewetting state of LM by manipulating the contact condition between the two interior interfaces.

To further show the utility of this phenomenon, we dewet the LM over surface topography under two scenarios. In the first scenario, the LM is physically spread across the surface. The strong mechanical tension of the metal prevents it from penetrating into the topographical recesses; thus it is in the Cassie–Baxter state (**Figure** **4**a). The film remains in this state until the contact angle (θ_*s*_) becomes below the equilibrium contact angle (θ_*E*,*s*_). To initiate the dewetting, we simply scratched the film with a stick. Dewetting commences and leaves behind the free‐standing oxide skin suspended across the structured surface (Figure 4b). The oxide skin is apparent since it fractured/wrinkled during the vacuum process needed for SEM imaging (Figure 4c). As shown in Figure 4d, the free‐standing oxide skin smoothly covers the surface and is suspended by multiple cylindrical PDMS posts. Current fabrication methods such as micro‐electromechanical system (MENS) fabrication,^[^ ^27^ ^]^ or capillary transfer^[^ ^28^ ^]^ are difficult to use for forming free‐standing nanomembranes. In contrast, this dewetting method is a relatively simple method to fabricate free‐standing nanomembrane (≈6 nm) on a solid substrate.

In the second scenario, we first filled the LM into a topographically patterned PDMS substrate by a method described elsewhere.^[^ ^29^ ^]^ This state resembles the Wenzel state (Figure 4e). The geometry of the channel is designed to prevent LM dewetting.^[^ ^29^ ^]^ By dewetting the LM film by scratching, we can leave behind the metal only in the channels This is a facile method to make a multilayered LM stretchable circuit. Here, we formed a near‐field communication (NFC) coil that was used to wirelessly power a LED (Figure 4g,h). The device works even while being stretched (Figure 4h).

## 3 Discussion and Conclusion

This paper reports that the interior of the native oxide layer that forms on liquid metals significantly lowers the interfacial energy of the metal from 724 to ≈10 mN m^−1^. Like molecular surfactants, the oxide skin is asymmetric: the exterior of oxides forms hydroxyl groups, but the interior of the oxide skin–that is, the part in contact with the metal–forms sub‐oxides that can form metallic bonds with the liquid metal. These bonds decrease the interfacial energy at the metal‐oxide interface (γ_*ol*_) to ≈10 mN m^−1^, which is a much larger decrease in tension than is possible using conventional surfactants. To date, this value of interfacial energy was challenging to measure due to complications arising from the solid oxide that encases liquid metal.

This drop in tension cannot be explained solely by dispersive interactions; if we assume only dispersive interactions are present at the interface, the γ_*ol*_ should be at least 569 mN m^−1^, an enormous value that is inconsistent with the interfacial behavior of LMs (Supporting Information, Calculation of interfacial energy and Hamaker constant of materials). Hence, we suggest that the short‐range metallic bonding between the LM and the interior of the oxide is responsible for the low interfacial energy. This low value is noteworthy because conventional surfactants can only modestly lower the surface energy of metals.^[^ ^12a^ ^]^ In contrast to molecular surfactants, the oxide forms spontaneously via reaction with air without the need to synthesize or externally add surfactant to a liquid.

In addition to significantly lowering the interfacial energy of metals, the exterior of the metal oxide has a high dispersive surface energy component compared to organic materials.^[^ ^30^ ^]^ Thus, the oxide facilitates strong adhesion with both polar and nonpolar solid surfaces (Figure 1a,b). Attempts to remove liquid metal from solid substrates usually cause the oxide to break at the air‐oxide interface, rather than detach from the substrate. The lack of liquid residue around the exterior of the drop in Figure 2d suggests that the oxide may break near where the air‐oxide interface meets the substrate.

Why is the interfacial energy between the metal and the interior of the oxide so low? Gallium is most stable in the Ga^3+^ state and therefore should form Ga_2_O_3_. However, previous spectroscopic characterization suggests the native oxide is incompletely oxidized and thus forms “sub‐oxides”: Ga_2_O_x_, in which x<3.^[^ ^26^ ^]^ Likewise, depth‐resolved XPS, and X‐ray reflectometry suggest that Ga^+^ exists closer to the metal interface, and segregates at the interface.^[^ ^5^ ^]^ This highly concentrated Ga^+^ of native oxide at the interface provides metallic characteristics to the suboxide.^[^ ^6^ ^]^ This can enable metallic bonding that lowers the energy at the metal‐oxide interface.

While we focused on liquid Ga because of its convenient temperature window, the above discussion implies that native oxides on metals should have similar properties if they have metallic suboxide at the interface. For example, the aluminum oxide (Al_2_O_3_) that forms on liquid (or solid) Al also has an Al‐terminated interior.^[^ ^31^ ^]^ Similar to Ga, this atomic layer of Al at the interface originates from the Al suboxide such as Al_2_O and Al‐O and it shows metallic characteristics.^[^ ^32^ ^]^ Titanium (Ti),^[^ ^33^ ^]^ indium (In),^[^ ^34^ ^]^ molybdenum (Mo),^[^ ^35^ ^]^ or other metals also produce metallic suboxide at the interface,^[^ ^36^ ^]^ and potentially serve as interfacial energy stabilizing oxide skin for LM of itself or LM alloys. In addition, metals that do not produce metallic suboxide can be stabilized by introducing impurities and foreign oxide. For instance, dissolved Ti in a noble molten metal such as Au (forms titanium suboxide at the interface, thereby allowing it to adhere to surfaces it would normally not wet, such as Al_2_O_3_.^[^ ^37^ ^]^

The low interfacial energy is useful for stabilizing LM films and other structures since the metal wets its interior oxide. Yet, films and droplets of the metal can be destabilized by contacting the interior layers of the oxide, resulting in dewetting of the LM. The LM continues to dewet from the interior oxide “sac” until it achieves an equilibrium contact angle. The unique dewetting process is useful for depositing oxides on surfaces at ambient conditions (Figure S9 and Movie S3, Supporting Information), creating free‐standing oxide nanomembranes (1–5 nm) across topography (Figure 4b–d), selective LM dewetting on open channel structure^[^ ^29^ ^]^ (Figure 4f), or even generating bubbles encased by oxide nanomembranes at room temperature (Figure 3). According to the interfacial values measured here, if the contact angle of LM in the “sac” of oxide is above the equilibrium contact angle (≈18°), the LM structure is stable. This is consistent with liquid metal becoming unstable in its on‐oxide “sac” during receding measurements when the receding angle falls below ≈18°^[^ ^38^ ^]^ (Movie S4, Supporting Information).

The ability to deposit oxides on surfaces at ambient conditions is much simpler and faster relative to current methods to deposit oxides, such as atomic layer deposition, sputtering, or pulsed laser deposition, which use slow and demanding processes (e.g., vacuum processing). Furthermore, the ability to fabricate nanomembranes on a suitable growth substrate and transfer them to a donor substrate is non‐trivial. In contrast, the method here only requires simple dewetting on a surface. Moreover, Figure 3 implies even mm‐scale large‐area free‐standing nanomembranes can be fabricated.

The findings here suggest that native oxides of metals could potentially serve as an unprecedented surfactant to help stabilize the contact between LM and non‐metals, such as fillers (e.g., MXenes, diamonds, graphite, silicon carbide), liquids, or even air.^[^ ^39^ ^]^ Therefore, it is important for engineering composites. It will help to explain and enable new methods to achieve 2D–3D patterning of metals,^[^ ^29^, ^40^ ^]^ deposition of 2D materials,^[^ ^41^ ^]^ and stable metal/non‐metal composite materials.^[^ ^42^ ^]^ Therefore, the findings here provide fundamental insights into the behavior of molten metals with oxides and establish native oxide as a new class of useful “surfactant”.

## 4 Experimental Section

### Material

Gallium (Indalloy 14), and EGaIn (Indalloy 60) were purchased from Indium Corporation (NY, USA). In (4N) and Sn (4N) were purchased from VTM Co. (Vacuum Thin Film Materials, South Korea). Galinstan was prepared by adding solid indium and tin beads into preheated liquid gallium. The gallium was heated to 60 °C and added In (21.5 wt.%) and Sn (10 wt.%) under 50 rpm of magnetic stirring in the argon atmosphere for more than 48 h until no lump of In and Sn was observed. Sylgard 184 was purchased from Dow Corning Co. (MI, USA). Sodium dodecyl sulfate was purchased from Sigma–Aldrich (Merck Kagan, Germany), and Glass balls (3 mm diameter) were purchased from Paul Marienfeld GmbH & Co. KG (Germany). Fused silica wafers were purchased from University Wafer Co. (MA, USA).

### JKR Method and Snap‐in Force‐Distance Profile Measurement

PDMS lens fabrication started with a glass ball (3 mm diameter) attached at the tip of a glass capillary with super glue. It was dipped in Sylgard 184 (20:1 mixing ratio between the base polymer and curing agent) and cured at 80 °C for 2 days. The PDMS spontaneously forms a spherical cap shape on the glass ball. The average radius of curvature which is measured by optical microscopy and imageJ software was 1.09 ± 0.03 mm. It was connected to a force sensor (GSO‐10 Transducer Technique, Temecula, CA, US) fixed on a linear actuator of a goniometer (Phoenix‐MT(T), Surface Electro‐Optics, South Korea). It was gently touched on the oxide skin of each liquid metal puddle covering a fused silica wafer. As it approached the substrate, the dewetted contact area was monitored by an optical microscope. The JKR measurements of force were acquired after holding the lens statically in position for 5 min to allow the system to equilibrate. Once an individual measurement was taken, the lens was then moved slowly (180 nm s^−1^) to a new position to minimize any viscous effects. The force sensor was connected to a desktop computer via DAQ (NI9237, National instruments, Austin, TX, US), and was recorded with LabVIEW software (National Instruments, Austin, TX, US).

### Spontaneous Dewetting of an Air Bubble Inside Liquid Metal

A U‐shaped syringe filled with air was immersed in a liquid metal bath (8 cm × 8 cm × 3 cm) with a depth of 4 mm. 100 µl of air was injected into the bath. After dewetting was initiated, it was left for more than 30 min without additional pumping to reach equilibrium. The contact angle recovery experiment was started from a bubble that reached equilibrium. The bubble was deflated every 5 s by 1.4 µl for 9 times. The images for each step were captured before each deflation step. After the deflation was over, the image was captured every 10 min to track the contact angle change.

### TEM Imaging

Samples were prepared in 1) an inert argon atmosphere for TEM imaging of the interface of gallium and a silicon wafer, and 2) air for imaging the interface of a droplet of gallium encased in native gallium oxide, placed on a silicon wafer. A liquid gallium droplet was dispensed on a silicon wafer (with a native oxide of SiO_2_). It was then compressed by a flat Teflon substrate to push the two materials together. Then solid gallium was brought into contact with the liquid gallium to nucleate the solidification of the liquid gallium when its temperature was below its melting point. The Teflon substrate was then removed gently to avoid disturbing the interface between the Ga and the substrate. A focused Ion Beam (FIB, Nova 600 Nanolab) was introduced to sample the wafer. TEM (JEOL 2010F) imaging was operated at 200 kV.

## Conflict of Interest

The authors declare no conflict of interest.

## Author Contributions

M.D.D. and W.J. performed conceptualization. W.J. and M.H.V. performed methodology. W.J., M.H.V., K.K., J.K., and S.K. performed the investigation. W.J. performed visualization. M.D.D. and T.K. acquired funding. M.D.D. and T.K. performed project administration. M.D.D. and T.K. performed supervision. W.J., M.D.D., and T.K. wrote the original draft. W.J., M.D.D., and T.K. wrote, reviewed, and edited the final manuscript.

## Supporting information



Supporting Information

Supplemental Movie 1

Supplemental Movie 2

Supplemental Movie 3

Supplemental Movie 4

Supplemental Movie 5

## Data Availability

The data that support the findings of this study are available from the corresponding author upon reasonable request.
