# Supplementary material for: Giant Decrease in Interfacial Energy of Liquid Metals by Native Oxides
Source: Adv Mater. 2024 Oct 10;36(48):2406783. doi: 10.1002/adma.202406783 (PMC11602690; doi:10.1002/adma.202406783)
Supplement: Supplementary file 1 — Supporting Information [file ADMA-36-2406783-s002.pdf]

ADVANCED MATERIALS

Supporting Information

for *Adv. Mater.*, DOI 10.1002/adma.202406783

Giant Decrease in Interfacial Energy of Liquid Metals by Native Oxides

*Woojin Jung, Man Hou Vong, Kiyoon Kwon, Jong Uk Kim, S. Joon Kwon, Tae-il Kim**
*and Michael D. Dickey**

Supporting Information

Giant Decrease in Interfacial Energy of Liquid Metals by Native Oxides

Woojin Jung[1], Man How Vong[2], Kiyoon Kwon[1], JongUk Kim[1], S. Joon Kwon[1], Tae-il Kim [1], Michael D. Dickey[2]**

W. Jung, K. Kwon, J. Kim, S. J. Kwon
School of Chemical Engineering,
Sungkyunkwan University (SKKU),
Suwon 16419, Korea

M. H. Vong
Department of Chemical and Biomolecular Engineering,
North Carolina State University (NCSU),
Raleigh, 27695, USA

[*] Prof. T. Kim
School of Chemical Engineering,
Sungkyunkwan University (SKKU),
Suwon 16419, Korea
Email: taeilkim@skku.edu

[*] Prof. M. D. Dickey
Department of Chemical and Biomolecular Engineering,
North Carolina State University (NCSU),
Raleigh, 27695, USA
Email: mddickey@ncsu.edu

Keywords: liquid metal, gallium, EGaIn, galinstan, interfacial energy

Supporting Text

Text S1. Calculation of interfacial energy and Hamaker constant of materials Surface energy is a sum of Gibbs free energy per unit area of every intermolecular interaction on the surface. Intermolecular interactions are divided into two categories: long-range and short-range interaction. Dispersive interaction which is described by Lifshitz theory of van der Waals interactions is long-range interaction occurring in the range of less than a few micrometers. This interaction is universal. All molecules and atoms show dispersive interaction. While other interactions such as metallic bonding (*m*), Lewis acid-base interaction, and hydrogen boding are short-range interactions occurring in the range of sub-nanometer [43]. In general, these interactions can sum to give the overall surface energy according to equation S1.

$$\gamma_i = \sum_j \gamma_i^j \qquad (S1)$$

in which i and j represents the interface of a semi-infinite material i and various intermolecular interactions, respectively. Equation S1 is also true when i represents an interface between two materials.

The dispersive interaction contribution to the surface energy of a semi-infinite material i in vacuum can be calculated with the equation S2 below. The material could be metal or oxide, for example.

$$\gamma^d = \frac{A_{ii}}{24\pi {l_0}^2} \qquad (S2)$$

Here, l_0 is dispersive separation distance, 0.165 nm, which is a universal constant for dispersive interfacial energy calculations, and A_{ii} is Hamaker constant of the material i [44].

The Hamaker constant of metals can be calculated using the plasma frequency (ω_p) [33, 45].

$$A_{11} = \frac{3kT}{4} + \frac{3h\omega_{p,1}}{32\pi\sqrt{2}} \qquad (S3)$$

The plasma frequency of gallium and EGaIn is reported elsewhere [46]. The Hamaker constant of gallium and EGaIn at room temperature is 3.34×10^{-19} J, and 2.96×10^{-19} J, respectively. Since $\gamma_l = \gamma^d + \gamma^m$, according to the equation S1, we can calculate γ^m by subtracting γ^d from the experimental value of materials. In Table S2, we summarize the Hamaker constant (*A*), the dispersive interaction component (γ^d), metallic bonding (γ^m), and total surface energy of each material (γ_l) in vacuum.

Text S2. Johnson-Kendal-Roberts (JKR) adhesion When two surfaces contact and form an interface, free energy changes due to the intermolecular interactions. This thermodynamic work per unit area is adhesion energy (W). The interfacial energy between material 1 and 2 is determined by the equation S4.

$$\gamma_{12} = \gamma_1 + \gamma_2 - W_{12} = \sum_j \gamma_1^j + \sum_j \gamma_2^j - \sum_j W_{12}^j \qquad (S4)$$

Especially, if only dispersive interaction is responsible for the adhesion energy ($W_{12} = W_{12}^d$), equation S5 gives excellent approximation to γ_{12} [48].

$$\gamma_{12} = \gamma_1 + \gamma_2 - W_{12}^d = \gamma_1 + \gamma_2 - 2\sqrt{\gamma_1^d \gamma_2^d} \qquad (S5)$$

If short-range interactions participate in the adhesion, W_{12} becomes larger than W_{12}^d except rare cases such as an interface between monopolar surfaces.

We used JKR (Johnson-Kendal-Roberts) adhesion theory to model the adhesion behavior between two interior oxide skins in the presence of liquid metal (i.e. a 'sac' of oxide encasing a volume of liquid metal), and thereby extract the oxide-liquid interfacial energy. The JKR adhesion theory is described as (*19-22*)

$$r^3 = \left(\frac{R}{K}\right)[F + 3\pi WR + \{6\pi WRF + (3\pi WR)^2\}^{0.5}] \quad (S6)$$

$$\frac{1}{K} = \frac{3}{4}\left\{\frac{(1 - \nu_{PDMS}{}^2)}{E_{PDMS}} + \frac{(1 - \nu_s{}^2)}{E_s}\right\} \quad (S7)$$

where $r, R, F, W,\ \nu_i$, and E_i is radius of contact area, radius of curvature of the PDMS lens, force acting on the lens, adhesion energy, Poisson's ratio, and elastic modulus, where the subscript i refers to either the PDMS lens or substrate (s). K is defined in Equation S7. Here, we assume that the effect of dispersive interaction of the PDMS lens and the substrate (here, fused silica) to the adhesion of the top and bottom oxide skin is sufficiently small to be neglected since the measurements were nearly identical with two very different substrates (fused silica and PDMS). Even though the oxide skin is extremely thin, it is strong enough to hold the heavy liquid metal inside of it. Thus, it could affect K. For this reason, we fit the relationship between F and r^3 using Equation S6 with using γ_{ol} and K as the fitting parameter. Note that we assume that the adhesion energy between the two oxide skins is $2\gamma_{ol}$ because the interfaces are identical.

Before we tested the oxide skin/LM interface, we tested PDMS in order to validate the JKR experiment. Figure S10 shows the result of the PDMS lens / PDMS substrate. From the fit to the data, we found the surface energy of the PDMS to be 20.1mN/m and the E_{PDMS} was 660 kPa (K = 588 kPa) [49]. We adopted ν_{PDMS} = 0.495 [49] which are well matched with our dynamic mechanical analysis (Figure S11, E_{PDMS}=643$\pm$2 kPa tensile mode, 1 Hz, 0.02% strain. Width, length, and thickness of the PDMS specimen size is 1 cm, 0.5 cm, and 0.1 cm, respectively.), and static tensile test (E_{PDMS}=619$\pm$61 kPa), and previous reports. The agreement of the data confirmed we were performing the analysis correctly.

We fit the data from each Ga-based LM alloys on a fused silica substrate. Each of the LMs were tested 3 times, more than 11 steps of approaching z direction for each trial (Figure 2 b). This reveals that the γ_{ol} of Ga, EGaIn, Galinstan is 9.1±0.8 mN/m, 10.3±1.7 mN/m, and 10.2±1.2 mN/m, respectively (K =1.31±0.05 MPa, R = 1.09±0.03 mm). As expected, the oxide skin affects the K. Based on the E_{PDMS} previously obtained, the calculated value of K should be 1.17 MPa (E_{SiO_2}=72.5 GPa, and ν_{SiO_2}=0.15). However, the experimental value was 0.14 MPa larger. That means the effective young's modulus of the PDMS lens covered with the oxide skin is 743 kPa which is 157 kPa larger than E_{PDMS}. We repeated the same test on PDMS substrate for the three LM alloys (Figure S5g-i). We observed a similar value of γ_{ol} and effective young's modulus of the oxide skin-covered-PDMS shows 775 kPa (K = 685±75.5kPa).

Equation S6 can be rearranged as Equation S8. By this equation, the different cases of JKR adhesion can be denoted in one plot to distinguish different adhesion energies.

$$\frac{r^3K}{3\pi R^2} = \left(\frac{F}{3\pi R} + W\right) + \sqrt{2W\frac{F}{3\pi R} + W^2} \quad (S8)$$

We measured adhesion energy between the exterior oxide skin and PDMS lens by the same JKR experiment (Figure S12.). The adhesion energy is 91.7 $\pm$ 4.8 mN/m. Since the surface energy of PDMS only has dispersive contribution, the adhesion energy only represents the dispersive contribution. Therefore, we can calculate the $\gamma_{o,ex}^d$ by the Equation S5, and $\gamma_{o,ex}^d = 100.3 \pm$ 10.5 mN/m. Likewise, we calculated the W_{ol}^d, and γ_{ol}^d by using the $\gamma_{o,ex}^d$ and γ_l^d of gallium and

EGaIn assuming there is no short-range interaction (metallic bonding), and summarized in Table S3.

γ_{ol} must be greater than $\gamma^m + \gamma_{ol}^d$ for each LM by equation S5, which is 569 mN/m and 326 mN/m for gallium and EGaIn, respectively. However, the experimental value was rather similar to γ_{ol}^d. It implies that the metallic bonding contributes to the adhesion of LM/oxide skin.

Text S3. Equilibrium liquid metal puddle Large volumes of liquid metal placed on a solid substrate form puddles rather than droplets due to gravity. At equilibrium, they show constant thickness as a consequence of a balance between the gravity and the effective interfacial energy (the value of which depends on whether the metal is coated with an oxide or if it is bare). The thickness of the puddle can be expressed by a simple equation below.

$$t_{E,s} = 2\lambda^{-1} \sin\frac{\theta_{E,s}}{2} \qquad (S9)$$

$$\lambda^{-1} = \sqrt{\frac{\gamma}{\rho g}} \qquad (S10)$$

Here, $t_{E,s}$, λ^{-1}, $\theta_{E,s}$, γ, ρ, and g are the thickness of liquid puddle at equilibrium, capillary length of the liquid, equilibrium contact angle, effective surface tension of the liquid, density of the liquid, and standard gravity (9.8 m/s^2). The subscript E and s represent equilibrium, and substrate, respectively.

Figure S13a illustrates a schematic of liquid metal puddle dewetting from within a sac of its own oxide. Approximately 2 ml of LM is dispensed on a flat substrate. Subsequent to this, we establish contact between LM and a slide glass. Due to the strong adhesion between the slide glass and LM, we can pull the LM until it makes large puddle (4 cm × 5 cm). To facilitate the dewetting, excess LM was carefully removed to ensure that the thickness of the puddle is less than 500 μm. Before it dewets its own skin, the thin LM film maintains a metastable state. However, when the top and bottom oxide skins contact by pressing them together, a new triple point forms and dewetting proceeds until it reaches an equilibrium state. As the liquid recedes within the oxide-sac, the top oxide skin collapses on the bottom oxide skin (Movie S3, S4, and S5). The metal continues to withdraw until it reaches an equilibrium contact angle ($\theta_{E,s}$, Figure S13b). This LM puddle exhibits two distinct reflection planes: reflection plane 1, and reflection plane 2, located on the surface of the puddle, and substrate, respectively. These planes generate mirror images of the syringe needle, thereby we can measure the distance between the planes. This distance is $t_{E,s}$ (Figure S13c). We measured the $\theta_{E,s}$, and $t_{E,s}$ for liquid gallium, eutectic gallium indium (EGaIn), and Galinstan. All gallium-based liquid metals show similar $\theta_{E,s}$, and $t_{E,s}$, which are the range of 17-19°, and 780~820 μm (Figure S13d, e). Based on these values, we calculated the tension in the oxide by using Equation S9 and S10 substituting $\gamma_{ol} + \sigma_E$ into γ. The values are the range of 340~410 mN/m (Figure S13f), which is in excellent agreement with past rheological measurements of the surface yield stress of the oxide [2, 25].

Although we experimentally obtained the $\theta_{E,s}$, this can be also predicted by the Young's equation (Figure S13a) with the σ_E experimentally obtained from the Figure S7b). The predicted value was 18.1°, 17.7°, and 17.4° for gallium, EGaIn, and Galinstan, respectively. Likewise, the $\theta_{E,b}$ can be predicted theoretically in accordance with the force balance of Figure 3a.

Text S4. Validity of the Wilhelmy plate method to measure tension We made the following assumption to adopt the Wilhelmy plate method: we assume that the tension of the oxide skin of liquid metal puddle at equilibrium after dewetting could be considered a liquid-like surface tension when the strain induced by the external disturbance is negligible. This assumption could be divided into two: (1) The tension is constant and isotropic. (2) The tension before and after adhesion of the skin on the PDMS lens is constant since the contact area is negligible compared to the scale of the puddle. The profile of the meniscus around a cylinder is represented as below equation [50].

$$z \sim c\left(-\ln\epsilon + \ln 4 - \delta - \ln\left(x + (x^2 - c^2)^{\frac{1}{2}}\right)\right) \qquad (S11)$$

c, ϵ^2, and δ represents catenoid coefficient, Bond number ($Bo = \epsilon^2 = \rho g {r_0}^2/\gamma$, ρ is density of liquid, g is gravity, r_0 is radius of triple line, and γ is surface tension), and Euler-Mascheroni constant (0.57721⋯). z is zero at the initial LM surface (Figure S14). Although the solid surface of our system is spherical shape, the equation is still valid, because the meniscus is independent of the solid angle. We fit the meniscus in the equation S11 as shown in Figure S15.

Indeed, the fitting coefficient ϵ contains information of the tension acting on the meniscus. Therefore, we can calculate the tension without measuring the force if we know the position of z=0 (Fig. S18). However, we calculated it from the slope, force, and radius to minimize uncertainty. The edge obtained from image processing has 3-4 pixels width. Therefore, there is at least ± 1 pixel of the uncertainty. Figure S15 shows an example of the Galinstan meniscus. The tension calculated (σ_E) from the measured force was 399 mN/m from the picture. Note that this value agrees with the σ_E calculated from the equilibrium puddle thickness. The distance the PDMS lens traveled until contact was 41 ± 1 pixels. The pixel #3 is the 41st pixel, and the #2 and #4 is 40th pixel, 42nd pixel, respectively. We calculated the tension for each pixel as shown in the Figure S16. Even if the value of pixel #3 (420 mN/m) is close to the value from the measured force, uncertainty of the tension fell within the range of 320~551 mN/m.

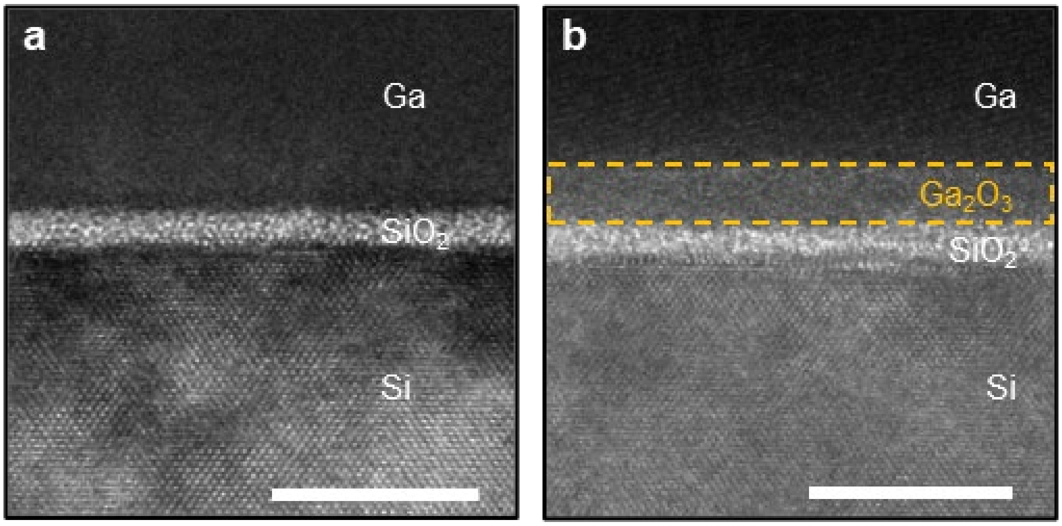

Figure S1. Interface of gallium in inert atmosphere, and air. (a, b) TEM image of the interface between gallium and a silicon wafer. In inert atmosphere, gallium directly contacts with the wafer (a), while the native oxide skin (Ga_2O_3) of gallium contacts with the wafer (b) in air. Scale bars (a, b) 5 nm.

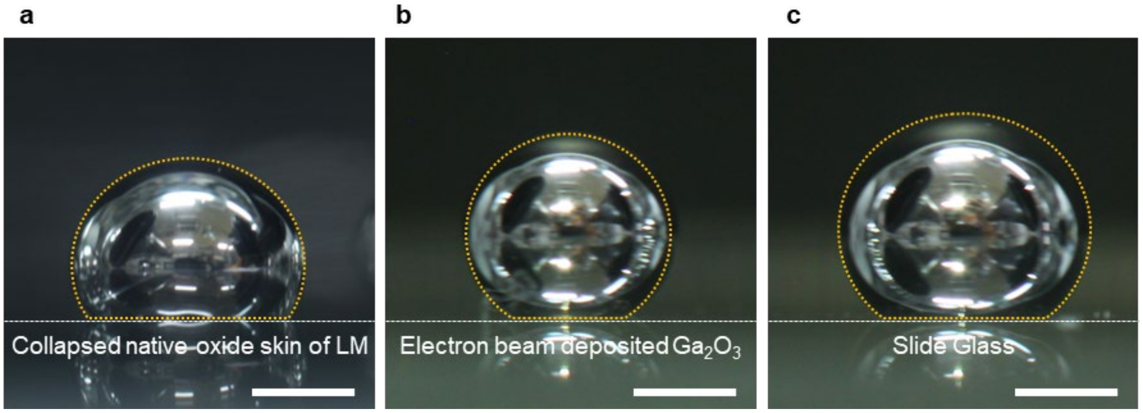

Figure S2 LM does not wet the exterior of various oxide substrates. LM droplet on native oxide skin on LM collapsed on a slide glass (a), Electron beam deposited gallium oxide on a slide glass (b), and a pristine slide glass (c). Scale bars = 1 mm.

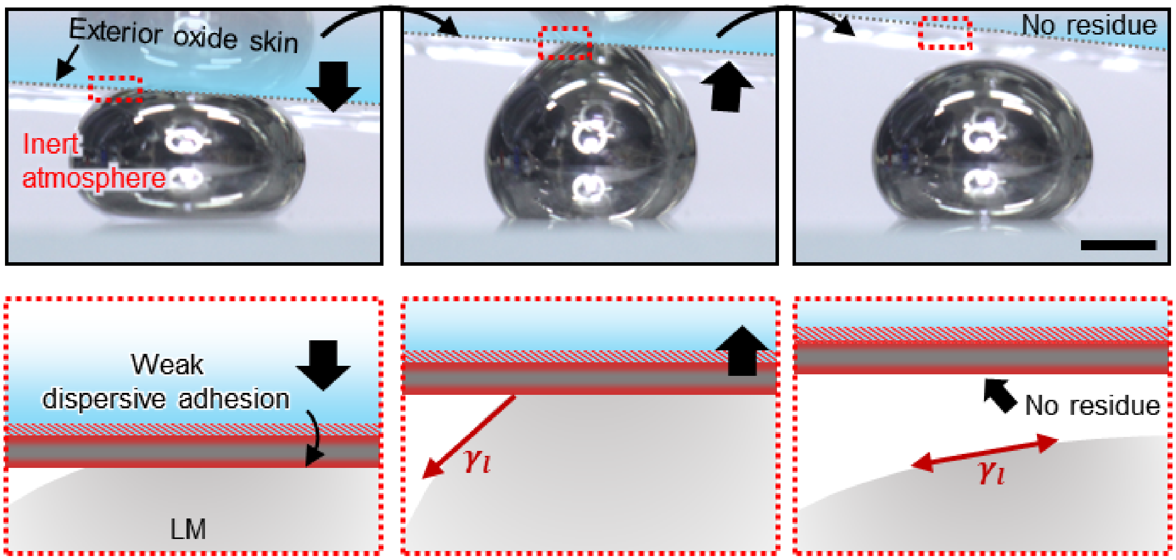

Figure S3. Adhesion behavior of LM to exterior of a native oxide skin in an inert atmosphere. The exterior oxide skin is deposited on the upper slide glass with the same dewetting process described in supplementary text, equilibrium liquid metal puddle. It is then brought into contact with a droplet of bare LM in a glove box. Consequently, both the interface of the oxide skin that contacts the LM is polar, not metallic. Scale bar = 5 mm.

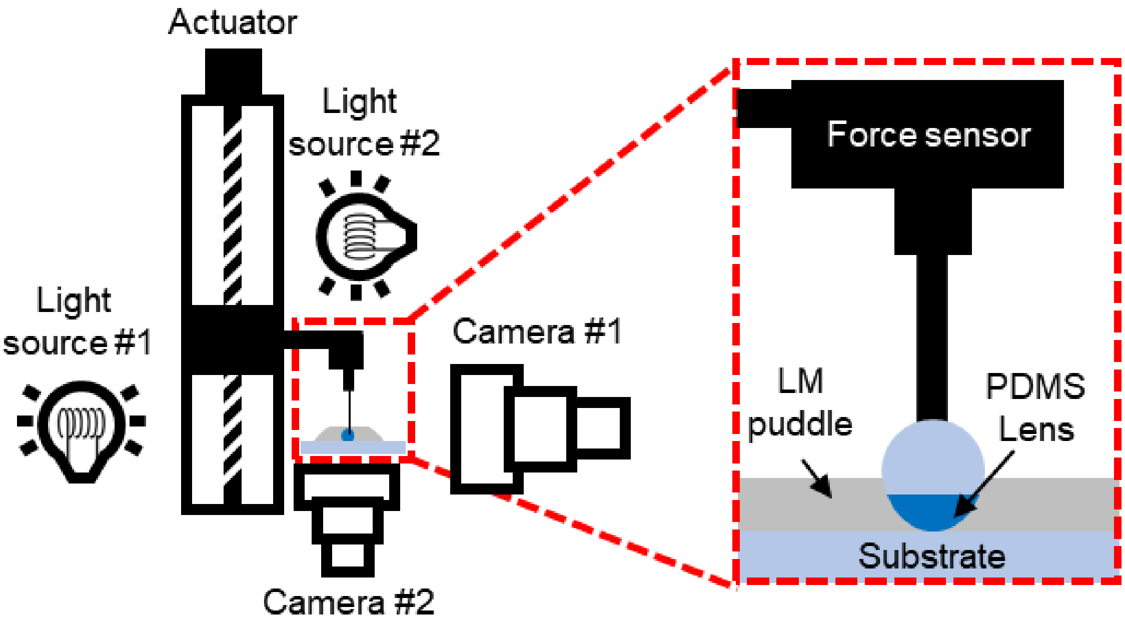

Figure S4. Experimental set-up for JKR measurements of LM. As the PDMS lens approaches the substrate, the exterior of the oxide skin on the liquid metal puddle intimately conforms to the PDMS lens, effectively creating a lens with conformal 'interior' oxide facing into the remainder of the LM puddle. We lowered the oxide-coated PDMS lens to the substrate at a speed of 180 nm/s until the PDMS lens (radius = 1.09±0.03 mm) touched the bottom LM/oxide skin interface. This process effectively brings the two interior oxide layers (one from the air interface and one at the silica substrate) in contact.

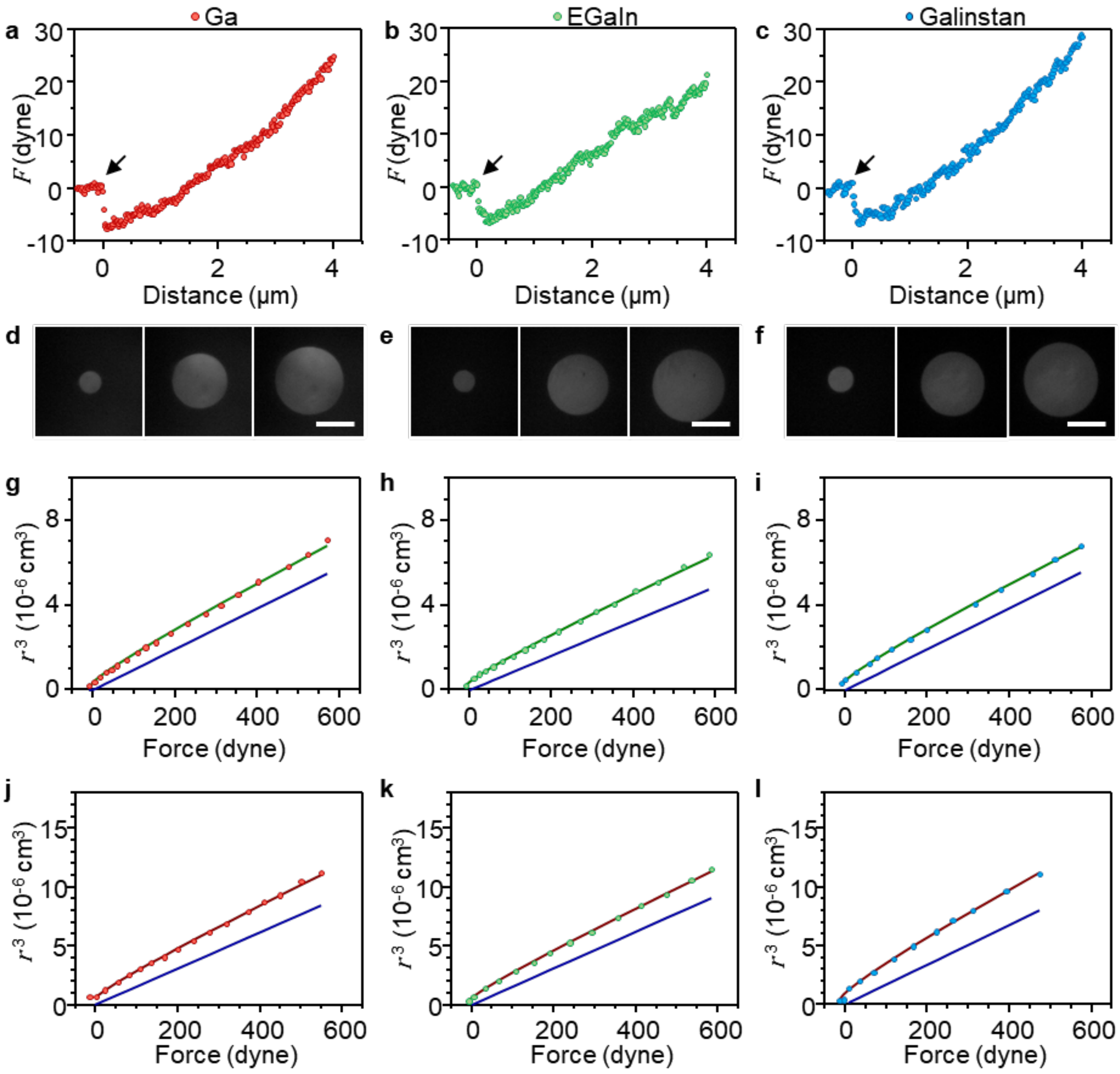

Figure S5. JKR experimental data. (a-c) Snap-in force measurement of three different LMs. Black arrow denotes onset of the snap-in at distance = 0 for each LM. (d-f) Micrographs taken from below the substrate indicating an increased contact area between the PDMS lens and substrate by pressing the lens further toward the substrate of gallium (d), EGaIn (e), and Galinstan (f). (g-l) r^3 versus F plot of JKR experiment on fused silica substrate (g-i) than is representative plot for each LM and PDMS substrate (j-l). Each plot was fitted by Equation (S6). Blue straight line represents the theoretical line of Hertz contact when the adhesion energy is zero. γ_{ol} of Ga, EGaIn, Galinstan on PDMS substrate is 7.8 mN/m, 10.0 mN/m, and 13.1 mN/m, respectively. There are effectively three columns in this figure: the left most is for Ga (red data points), the center is for EGaIn (green data points), and the right is for Galinstan (blue data points). Scale bar = 100 μm

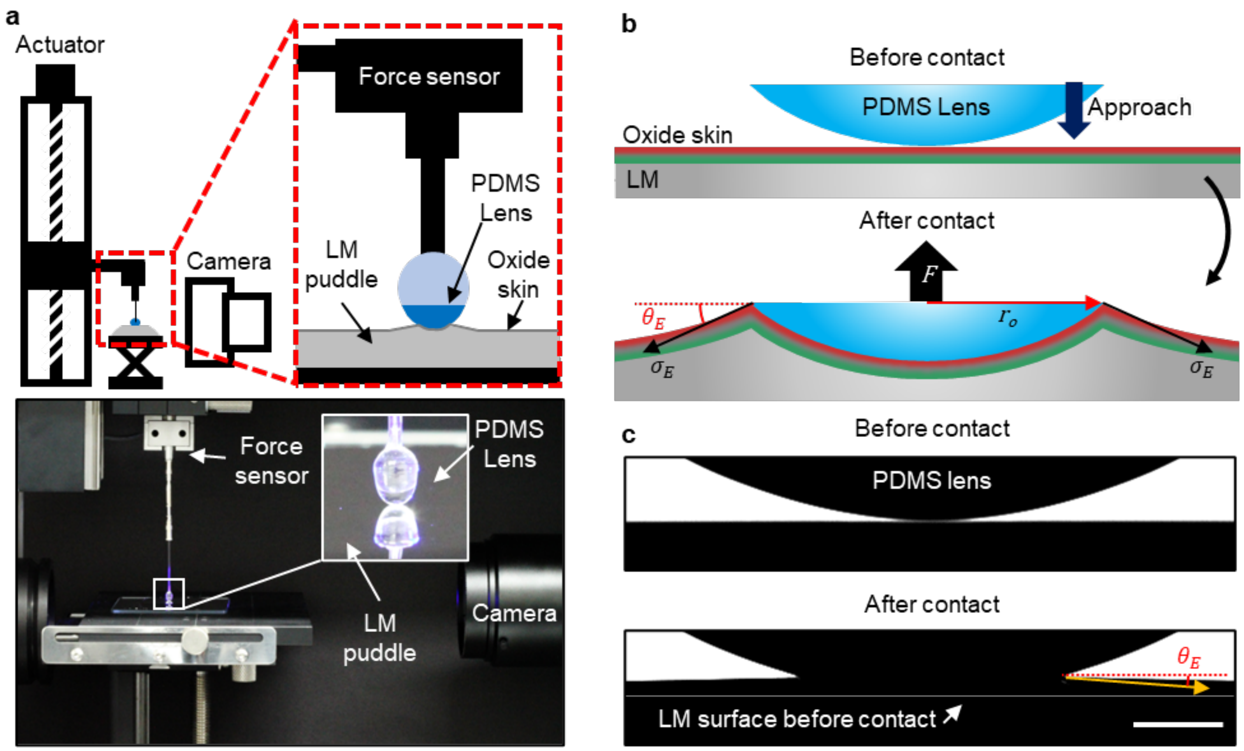

Figure S6. Experimental set-up for equilibrium tension (σ_E) measurement of oxide skin by Wilhelmy plate method. (a) Experimental set-up. A spherical lens of silicone (PDMS) is lowered vertically until it contacts the exterior of the oxide on a puddle of liquid metal (LM). The oxide adheres to the lens. As the lens is withdrawn from the puddle, a force is measured to estimate the tension of the oxide. (b) Schematic of a Wilhelmy plate method. The asymmetry of the oxide skin is denoted as red (dispersive, polar), and green (dispersive, metallic). (c) Profile image of LM (gallium) before contact (top) and after contact (bottom) with a PDMS lens. Scale bar (c) = 200 μm.

To obtain the tension, we formed a puddle of liquid metal (Supporting Text, Equilibrium liquid metal puddle). When a PDMS lens gently contacts a thin, smooth puddle of liquid metal (Figure S6b), the oxide-coated metal 'snaps' to the PDMS surface and exerts a downward force (F) that can be measured. We performed the measurements for three LM alloys (Movie S1). When contact occurred, the oxide-coated metal rose for several minutes to conform against the stationary lens and reached an equilibrium state [23a]. The force of the interface pulls down the PDMS lens [51]. The vertical force (F) is exerted by the vertical component of the equilibrium tension (σ_E) of the interface on the PDMS lens multiplied by the peripheral length ($2\pi r_o$) of the adhesion area,

$$\sigma_E \sin\theta_E = \frac{F}{2\pi r_o} \qquad (S12)$$

where θ_E is the equilibrium angle of the oxide skin relative to the horizon and r_o is the radius of adhesion area. To obtain θ_E, we fit the shape of the interface (Supporting Text, Validity of the Wilhelmy plate method to measure tension) and found the slope at the oxide-PDMS-air contact

line. The angle (θ_E) along with the measured force (F) and radius (r_o) were within a consistent range (Figure S7a).

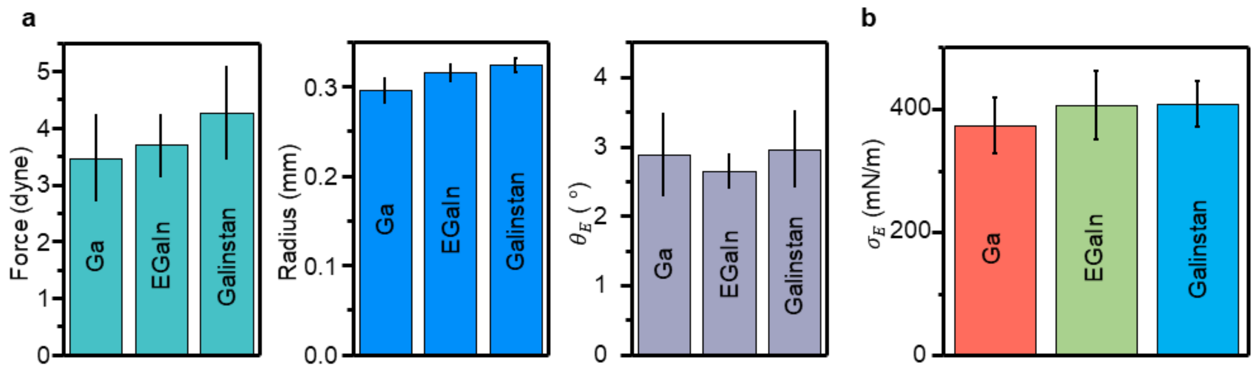

Figure S7. Experimental result of equilibrium tension (σ_E) of oxide skin (a) Force, contact angle (θ_E), and contact radius for each liquid metal obtained by Wilhelmy method. The angle (θ_E) along with the measured force (F) and radius (r_o) were within a consistent range of 2.5~3.0°, 3.4~4.2 dynes, and 290~330 μm. (b) Calculated tension of the oxide skin of each liquid metal.

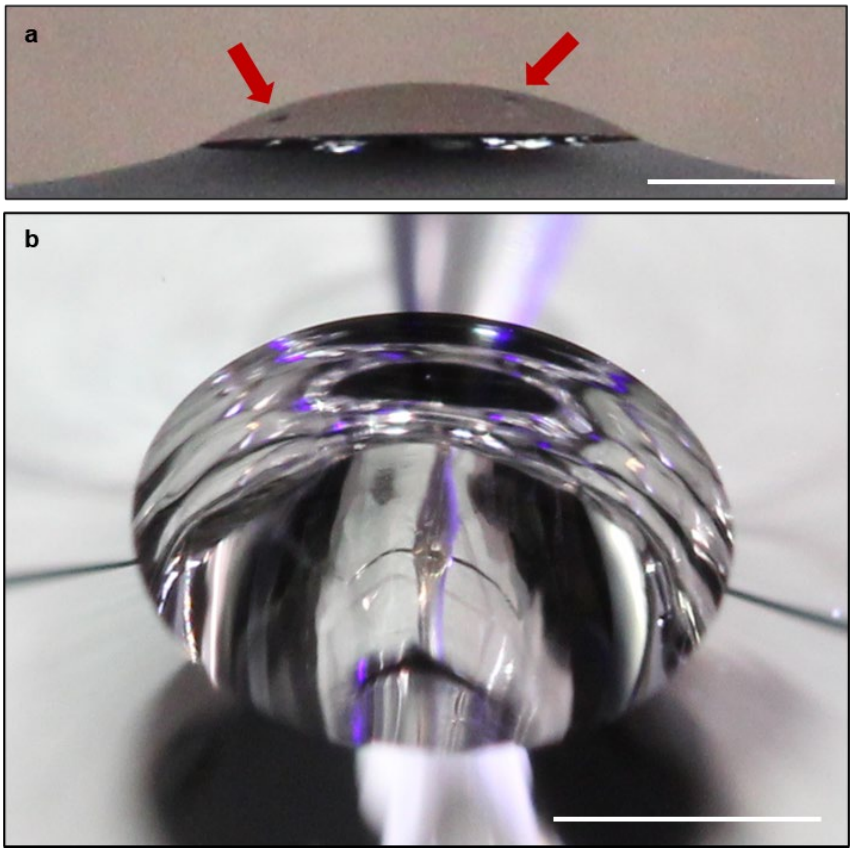

Figure S8. Side view (a) and top view (b) of an air bubble encased in oxide at the surface of a bath of liquid metal. (a) An air bubble delivered into a bath of liquid metal results in an oxide-encased bubble. The oxide is presumably only a few nm thick. Red arrows draw attention to small LM droplets trapped by the nanomembrane of the oxide skin. (b) A cavity inside of a bubble is clearly seen in the picture. The highly reflective metal surface is difficult to image, but it is apparent from the image that it is serving as an optical lens due to the meniscus shape of the metal inside the bubble. Scale bars = 2 mm.

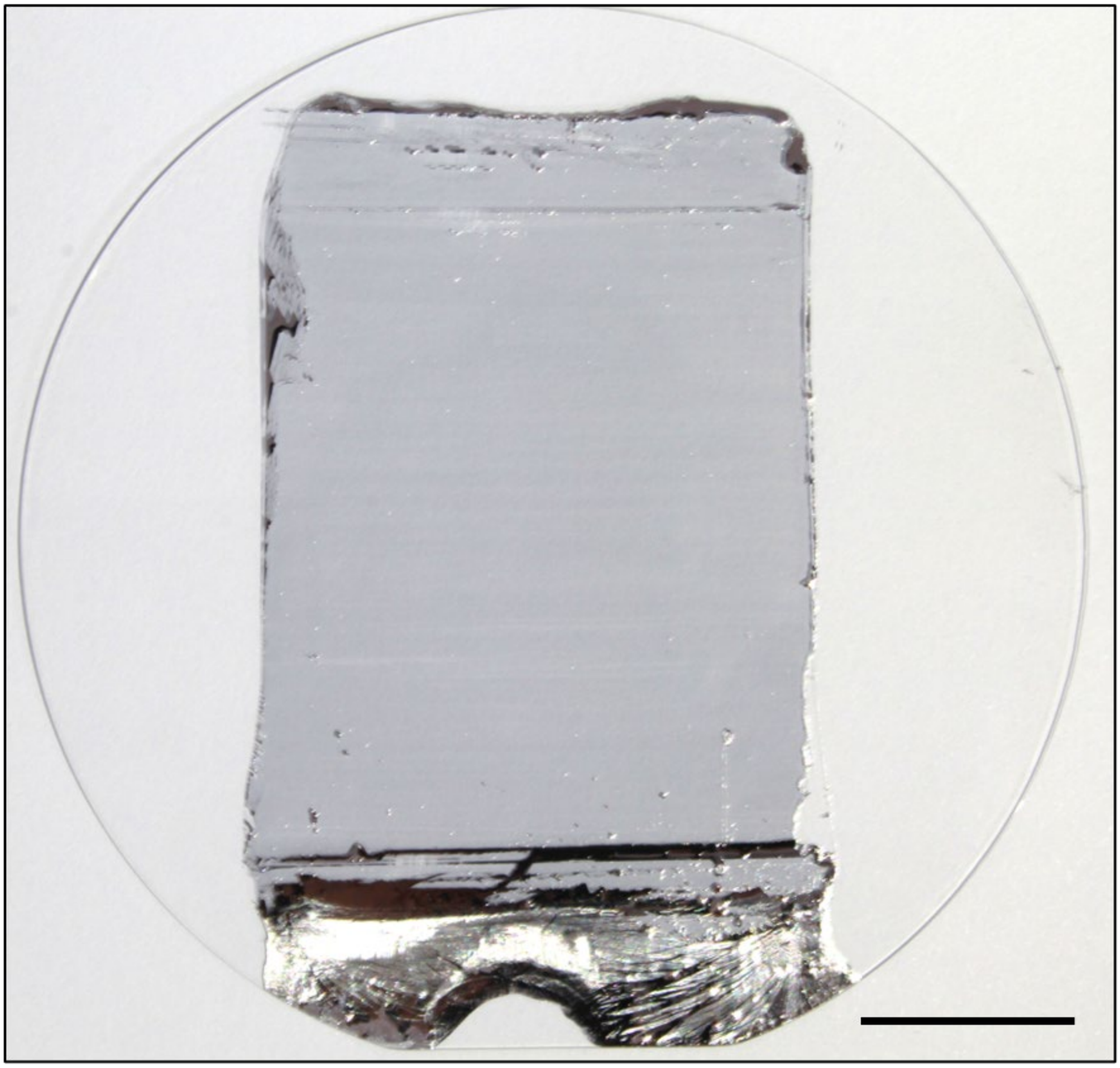

Figure S9. Wafer-scale thin film Ga_2O_3 deposition on 4-inch fused silica wafer via dewetting of liquid metal inside of its own oxide-sac. Transparent dark area is Ga_2O_3. Ga_2O_3 was deposited by the method described in the supplementary text, equilibrium liquid metal puddle by starting at the top of the wafer (as imaged here) and drawn to the bottom of the wafer. Liquid metal residue is observed at the edges, possibly due to wrinkles of the oxide skin which prevents dewetting. Scale bars = 2 cm.

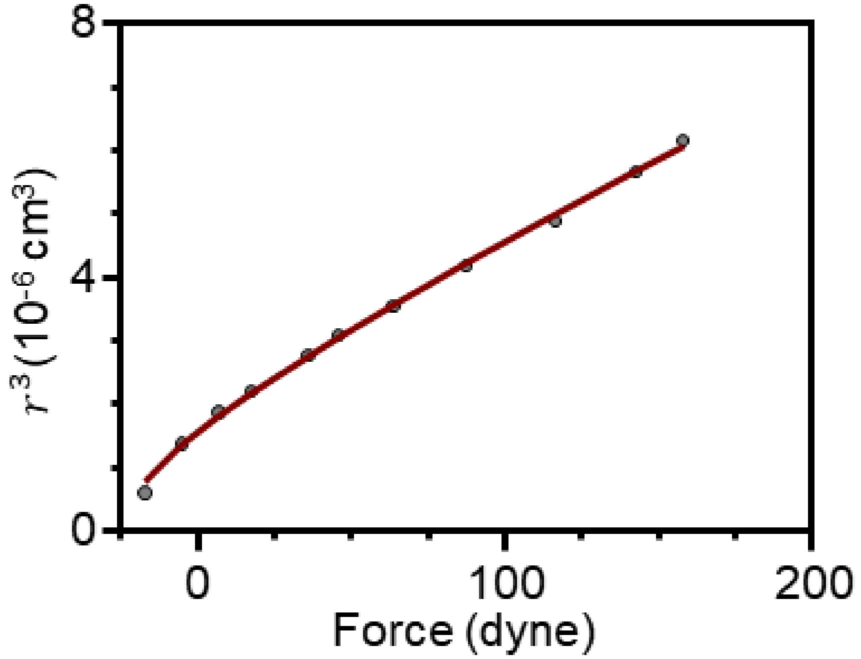

Figure S10. JKR experiment of PDMS lens/PDMS substrate without LM fitted by the equation *S*6. Experimental data is represented as grey dots, and the fitting curve is brown.

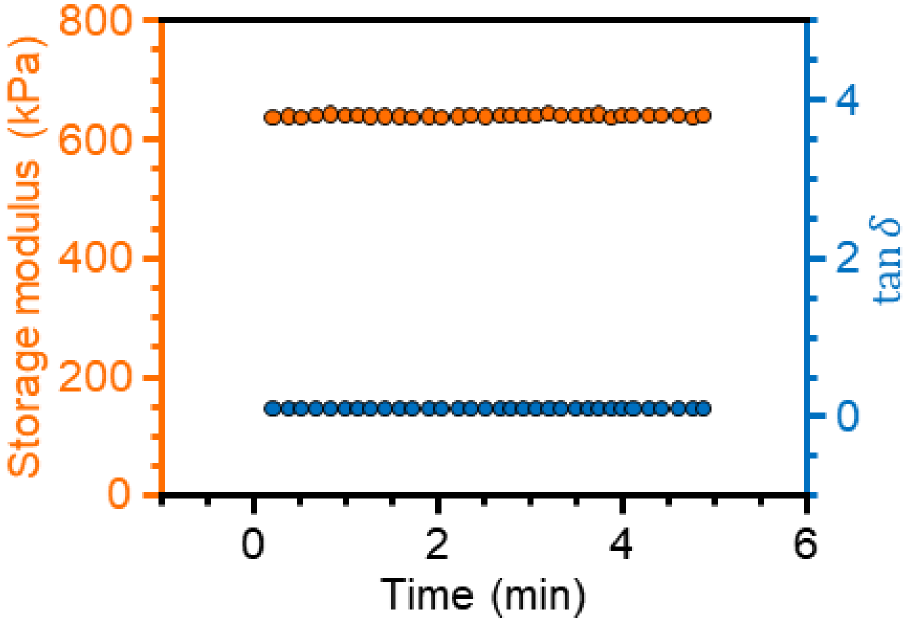

Figure S11. Dynamic mechanical analysis of PDMS at small strain (0.02%). The small tan δ clearly shows it is in elastic regime.

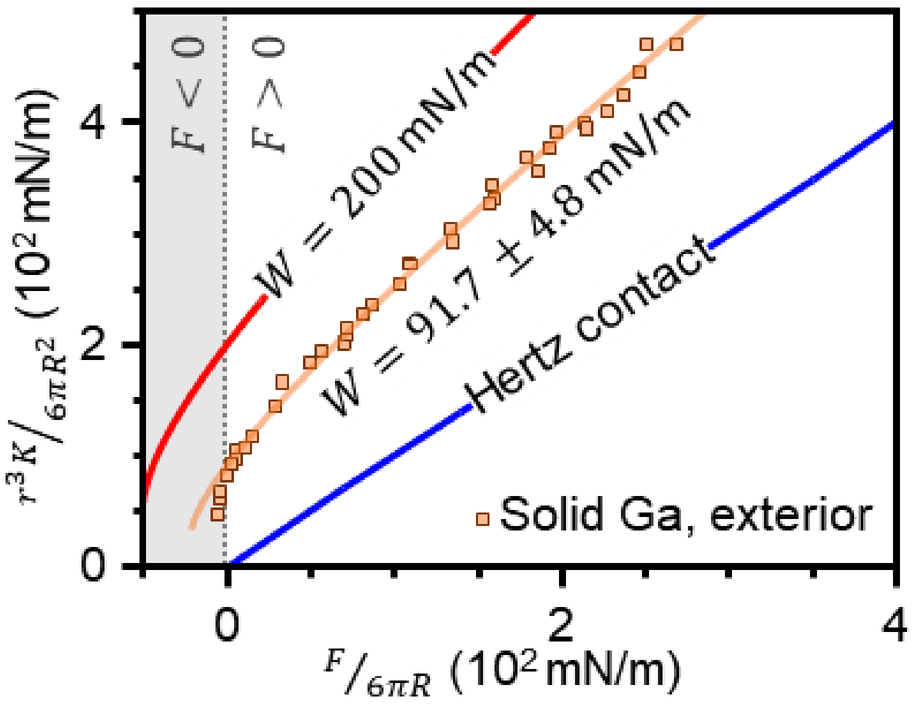

Figure S12. JKR experiments on the exterior of the oxide skin on solid Ga. The orange line represents the adhesion energy between the PDMS lens and oxide skin which is 91.7±4.8 mN/m.

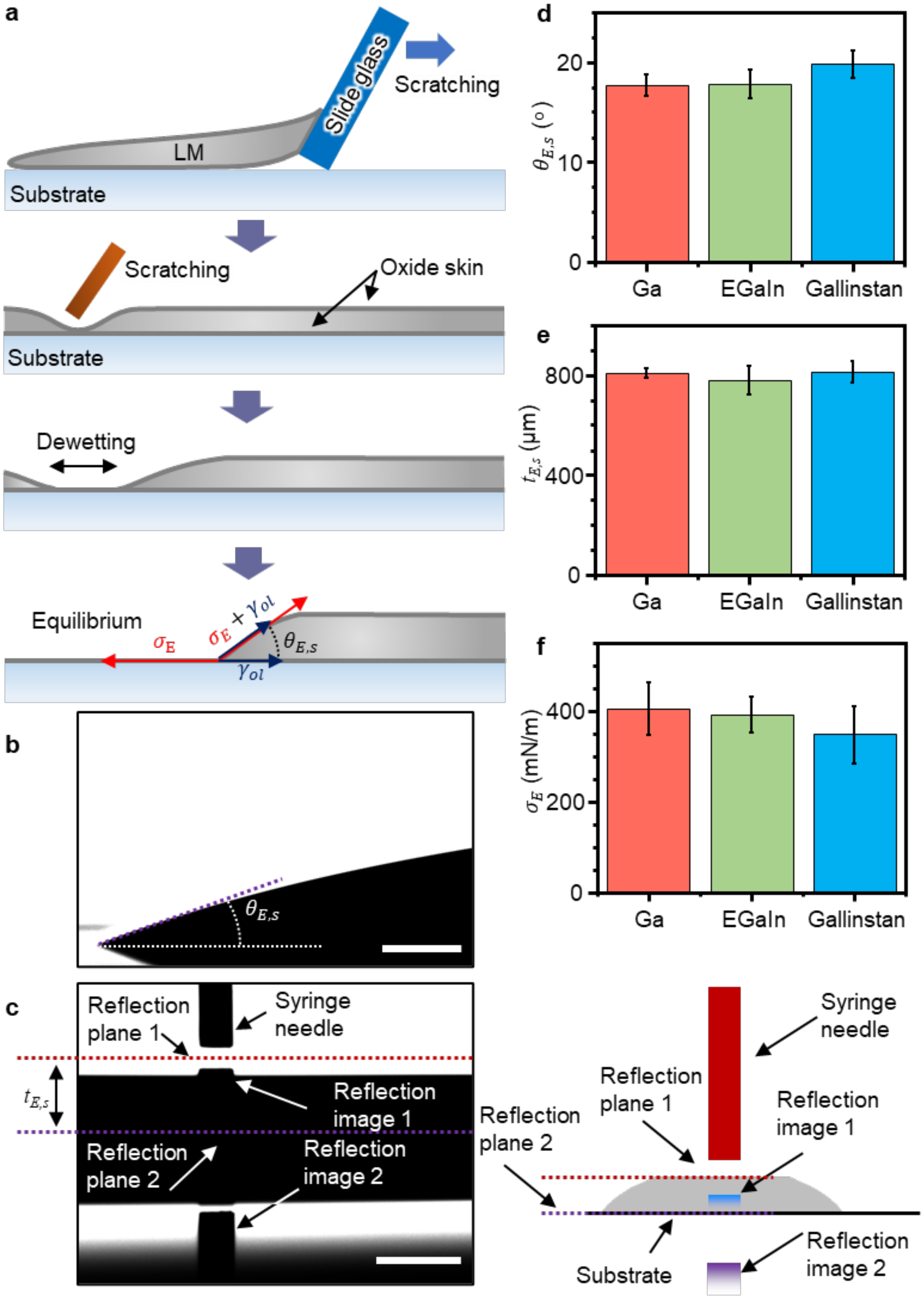

Figure S13. Equilibrium puddle geometry of each Ga-based LM alloys. (a) Schematic of liquid metal dewetting from within a sac of its own oxide. (b) Schematic illustrating measurement of the puddle thickness. (c-e) Contact angle (c), thickness (d), and tension of the oxide skin (e) of each Ga-based LM alloys. Scale bar = (b) 200 μm, (c) 1 mm.

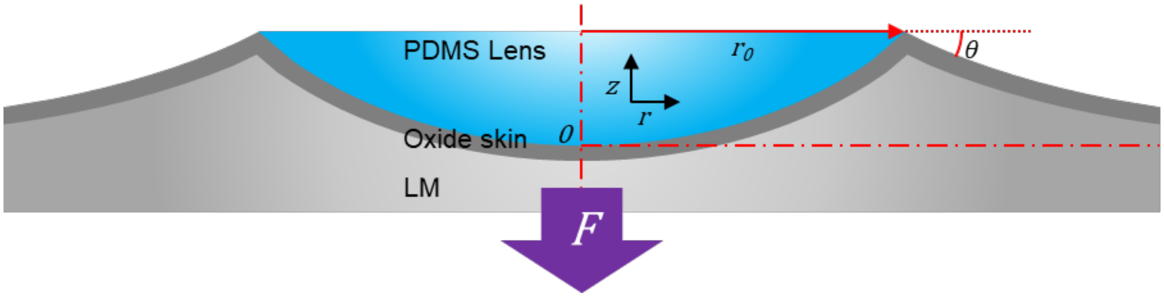

Figure S14. Schematic of Wilhelmy 'plate' method for measuring tension of oxide skin. Rather than a plate, here we use a spherical PDMS lens.

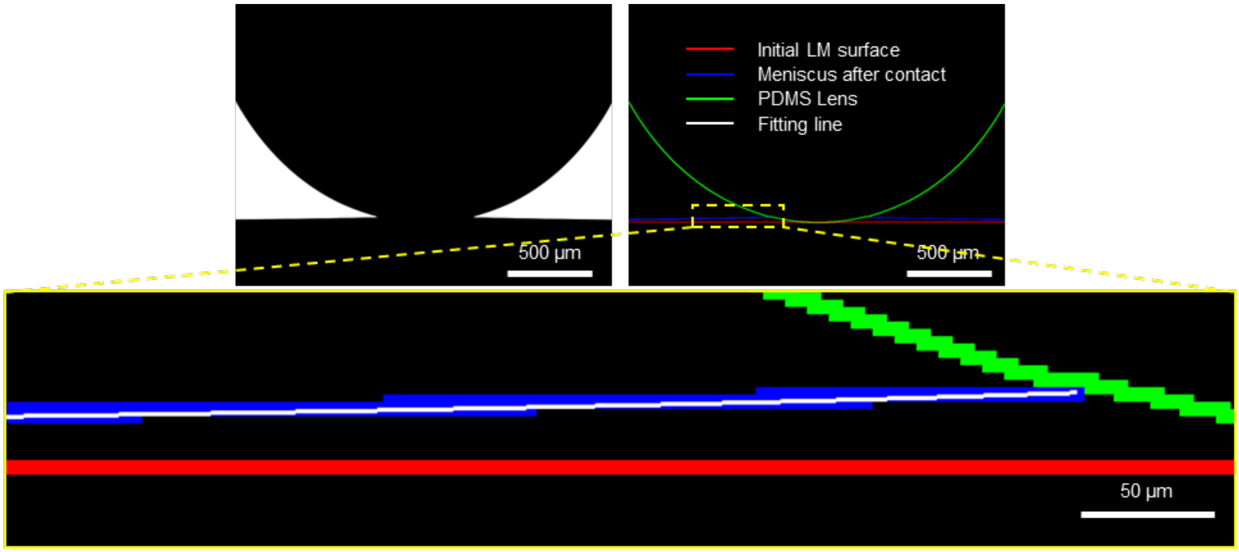

Figure S15. Theoretical fitting line of the meniscus profile (upper) and merged image (down). The profile of the meniscus was extracted by ImageJ software and fit by Equation S11 by MATLAB software. This fitting curve was converted into an image file and merged into the edge extracted image shown here.

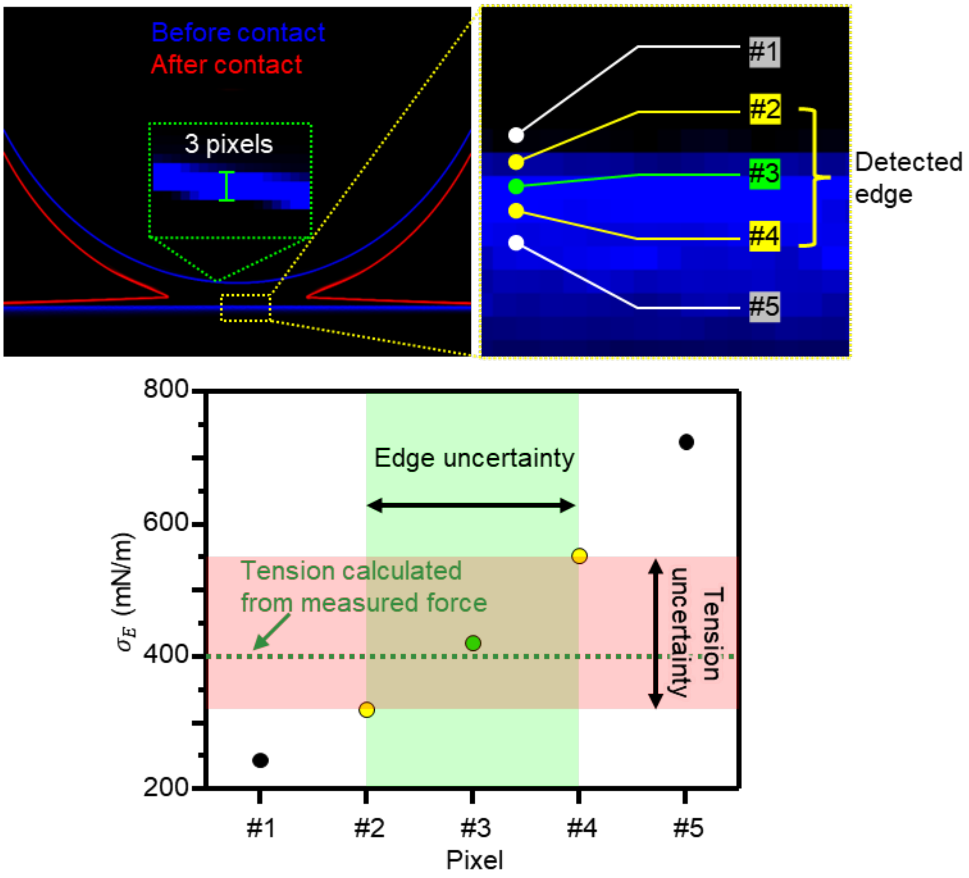

Figure S16. Uncertainty of the tension calculation in regards of meniscus profile.

Table S1. Interfacial property measurement of LM recently reported in the literature

Reference	γ_{eff}*	γ_{ol}	Decoupling	Method†
[13]	Y	N	N	CA
[53]	Y	N	N	CA
[54]	Y	N	N	CA
[55]	Y	N	N	CA
[56]	Y	N	N	CA
[57]	Y	N	N	CA
[58]	Y	N	N	CA
[26]	Y	N	N	CA
[25]	Y	N	N	CA
[2]	Y	N	N	R
[25]	Y	N	N	R, UTM
[26]	Y	N	N	R, CP
[59]	Y	N	N	R, PD
[60]	Y	N	N	PD
[9b]	Y	N	N	PD
[24a]	Y	N	N	DM
This work	Y	Y	Y	DM, WP, CA

*Effective tension of liquid metal.
†CA: contact angle, R: interfacial rheology, UTM: uniaxial tensile strength, CP: capillary pressure, PD: Pendant drop, DM: direct measurement, JKR: Johnson-Kendall-Roberts adhesion, WP: Wilhelmy plate.

Table S2. Hamaker constant and surface energy

	Ga	EGaIn	Fused silica (SiO_2)
A ($\times 10^{-19}$J)	3.34	2.96	0.65
γ_l (mN/m)	724	475*	N/A
γ^d(mN/m)	163	144	31.6
γ^m(mN/m)	561	321	0

*This value is estimate by plugging the measured interfacial tension of EGaIn in dilute HCl solution into equation S5 [52]. A and γ^d was adopted from the previous report [36].

Table S3. Calculated adhesion energy and interfacial energy of each interface.

	Ga/oxide skin	EGaIn/oxide skin
W_{12}^{d}(mN/m)	255.7	240.4
γ_{ol}^{d}(mN/m)	7.78	3.94

Table S4. Experimental values of interfacial properties of LMs

	Ga	EGaIn	Galinstan
γ_{ol}	9 mN/m	10.4mN/m	10.4 mN/m
σ_E	385.0±19.5mN/m	405.4±54.4mN/m	418.3±27.5mN/m
$\theta_{E,s}$	17.69±1.1	17.84±1.43	19.83±1.4
$\theta_{E,b}$	10.2±1.0	10.8±1.1	12.5±0.5

Movie S1.
Spontaneous adhesion of the native oxide skin of Gallium when PDMS lens contact to the oxide skin.

Movie S2.
Dewetting of LM air bubble from the LM between the surface oxide skin, and bubble oxide skin.

Movie S3.
Top view of spontaneous dewetting of LM on a flat solid substrate and collapsed oxide skin left behind. The white particle is stable although dewetting front of LM is passing by. This indicates that only the LM inside of its oxide-sac is moving, while the oxide skin is collapsing without following the dewetting front.

Movie S4.
Dewtting of metastable LM puddle. The thickness of LM puddle was below the equilibrium puddle thickness $t_{E,s}$. Scratching the puddle with wooden stick initiate the dewetting by creating the contact between the top and bottom oxide skin. Because the thickness is below $t_{E,s}$, we can expect the contact angle is below the equilibrium contact angle $\theta_{E,s}$,

Movie S5.
Side view of spontaneous dewetting of LM on a flat solid substrate.

References.

[43] a)M. K. Chaudhury, *Mater. Sci. Eng., R* **1996**, 16, 97; b)L.-H. Lee, *Langmuir* **1996**, 12, 1681; c)F. M. Fowkes, *Ind. Eng. Chem.* **1964**, 56, 40.

[44] J. N. Israelachvili, in *Intermolecular and Surface Forces (Third Edition)*, (Ed: J. N. Israelachvili), Academic Press, San Diego 2011.

[45] a)L. Bergström, *Adv. Colloid Interface Sci.* **1997**, 70, 125; b)L. Bergstrom, A. Meurk, H. Arwin, D. J. Rowcliffe, *J. Am. Ceram. Soc.* **1996**, 79, 339.

[46] a)D. Morales, N. A. Stoute, Z. Yu, D. E. Aspnes, M. D. Dickey, *Appl. Phys. Lett.* **2016**, 109; b)M. G. Blaber, C. J. Engel, S. R. C. Vivekchand, S. M. Lubin, T. W. Odom, G. C. Schatz, *Nano Lett.* **2012**, 12, 5275.

[47] C. J. van Oss, *Colloids Surf. Physicochem. Eng. Aspects* **1993**, 78, 1.

[48] a)L. Chen, E. Bonaccurso, M. E. R. Shanahan, *Langmuir* **2013**, 29, 1893; b)J. Vaicekauskaite, P. Mazurek, S. Vudayagiri, A. L. Skov, *J. Mater. Chem. C* **2020**, 8, 1273.

[49] A. Müller, M. C. Wapler, U. Wallrabe, *Soft Matter* **2019**, 15, 779.

[50] D. F. James, *J. Fluid Mech.* **1974**, 63, 657.

[51] a)D. Kumar, T. P. Russell, B. Davidovitch, N. Menon, *Nat. Mater.* **2020**, 19, 690; b)S. Chattopadhyay, Y. F. Huang, Y. J. Jen, A. Ganguly, K. H. Chen, L. C. Chen, *Mater. Sci. Eng., R* **2010**, 69, 1.

[52] a)D. Zrnic, D. S. Swatik, *J. Less-Common Met.* **1969**, 18, 67; b)J. E. B. Randles, D. J. Schiffrin, *Trans. Faraday Soc.* **1966**, 62, 2403.

[53]J.-E. Park, H. S. Kang, M. Koo, C. Park, *Adv. Mater.* **2020**, 32, 2002178.

[54]A. Hirsch, H. O. Michaud, A. P. Gerratt, S. de Mulatier, S. P. Lacour, *Adv. Mater.* **2016**, 28, 4507.

[55]A. Hirsch, S. P. Lacour, *Advanced Science* **2018**, 5, 1800256.

[56]R. K. Kramer, J. W. Boley, H. A. Stone, J. C. Weaver, R. J. Wood, *Langmuir* **2014**, 30, 533.

[57]Z. Chen, J. B. Lee, *ACS Applied Materials & Interfaces* **2019**, 11, 35488.

[58]Z. Ma, Q. Huang, Q. Xu, Q. Zhuang, X. Zhao, Y. Yang, H. Qiu, Z. Yang, C. Wang, Y. Chai, Z. Zheng, *Nat. Mater.* **2021**, 20, 859.

[59]Q. Xu, N. Oudalov, Q. Guo, H. M. Jaeger, E. Brown, *Phys. Fluids* **2012**, 24.

[60]D. Kim, P. Thissen, G. Viner, D.-W. Lee, W. Choi, Y. J. Chabal, J.-B. Lee, *ACS Applied Materials & Interfaces* **2013**, 5, 179.
